# Determinants of Brain Rhythm Burst Statistics

**DOI:** 10.1038/s41598-019-54444-z

**Published:** 2019-12-04

**Authors:** Arthur S. Powanwe, André Longtin

**Affiliations:** 1 Department of Physics, University of Ottawa, 150 Louis Pasteur, Ottawa, ON K1N6N5 Canada; 2 Department of Cellular and Molecular Medicine, 451 Smyth Road, Ottawa, ON K1H8M5 Canada; 3 Centre for Neural Dynamics, University of Ottawa, Ottawa, ON Canada

**Keywords:** Dynamical systems, Nonlinear phenomena

## Abstract

Brain rhythms recorded *in vivo*, such as gamma oscillations, are notoriously variable both in amplitude and frequency. They are characterized by transient epochs of higher amplitude known as bursts. It has been suggested that, despite their short-life and random occurrence, bursts in gamma and other rhythms can efficiently contribute to working memory or communication tasks. Abnormalities in bursts have also been associated with e.g. motor and psychiatric disorders. It is thus crucial to understand how single cell and connectivity parameters influence burst statistics and the corresponding brain states. To address this problem, we consider a generic stochastic recurrent network of Pyramidal Interneuron Network Gamma (PING) type. Using the stochastic averaging method, we derive dynamics for the phase and envelope of the amplitude process, and find that they depend on only two meta-parameters that combine all the model parameters. This allows us to identify an optimal parameter regime of healthy variability with similar statistics to those seen *in vivo*; in this regime, oscillations and bursts are supported by synaptic noise. The probability density for the rhythm’s envelope as well as the mean burst duration are then derived using first passage time analysis. Our analysis enables us to link burst attributes, such as duration and frequency content, to system parameters. Our general approach can be extended to different frequency bands, network topologies and extra populations. It provides the much needed insight into the biophysical determinants of rhythm burst statistics, and into what needs to be changed to correct rhythms with pathological statistics.

## Introduction

Fast oscillations in brain activity in the 30–100 Hz range, known as gamma rhythms, are observed across many brain regions and species, both *in vitro* and *in vivo*^1–4^. They occur either autonomously or are induced by external stimulation^5–9^. They have received much attention because of their proposed roles in several major neuronal processes like perception, cognition, binding, working memory or inter-areal communication^10–15^. To perform such tasks, it is generally believed that the gamma rhythm should be a coherent oscillation with relatively constant amplitude and frequency, in particular in theories where the oscillation acts as a clock signal^16,17^ with regular neuronal firing^18,19^.

However, several studies, focussing especially on gamma-range oscillations in monkey primary visual cortex, have reported that the rhythms are broadband rather than coherent, and exhibit transient epochs of elevated synchrony aptly termed “gamma bursts”. The underlying neuronal spiking activity is also quite irregular. These bursts of large oscillation amplitude alternate with epochs of almost no synchrony where the oscillation amplitude is low. The frequency shows a lot of variability, a consequence of the significant noisiness of the phase of the rhythm. Moreover, the occurrence times and durations of gamma bursts are random, making such rhythms closer to a broadband filtered noise than to a well-structured, almost periodic signal^20–22^.

Despite their stochasticity, such bursty rhythms have been shown to correlate better with the performance of certain tasks than more regular oscillations. Indeed, a recent study examined local field potentials (LFP) and spiking activity from the prefrontal cortex of monkeys performing a working memory task, and reported that working memory manifests itself through gamma bursts rather than sustained activity^23^. Another study measured neuronal activity in the entorhinal-hippocampal circuit while mice performed a reward-based spatial working memory task, and showed that gamma bursts contribute to the successful execution of the task^24^. A plausible role for such gamma bursts has recently been formulated computationally in the context of inter-areal synchronization and communication^25,26^.

There is currently no theory that links the properties of a network to those of the bursty rhythm. Here we provide such a general theory for a recurrent excitatory-inhibitory network. We show how the burst statistics relate to single cell and network parameters, and consequently to different regimes of oscillation. Apart from shedding light on how the bursts arise and can be used for neural computations, this theory provides the much-needed insight into how a system can be modified to rectify undesirable burst statistics associated with pathology. A useful framework to address the dynamics of such broadband oscillations is the amplitude-phase decomposition. The amplitude in this framework reflects the level of network synchronization, where weak values of the amplitude reflect little or no synchronization, whereas strong values reflect higher network synchronization. The phase, which depends on the amplitude to a good approximation, contains all the information about the temporal structure of the oscillation.

Amplitude-phase decompositions have been used in a number of computational studies to address phase synchronization^27^, inter-areal phase communication (generally known as communication through coherence or CTC)^26,28^ and more generally different types of cross-frequency-coupling (CFC) between brain circuits^29^. At the theoretical level, the search for such decompositions has been pursued over the last decades, particularly for oscillations of varying degree of coherence both at the single neuron and population levels^30–34^. Such studies belong to the broader effort to describe stochastic oscillations, sometimes called “quasi-cycles” in many areas of science including nonlinear chemical oscillators and population biology (see e.g.^35,36^). For the case of broadband gamma oscillations, recent studies^37–39^ have extracted from firing-rate-level descriptions of the network the generic dynamics for the slowly-evolving envelope of the rapidly-varying amplitude of the rhythm. This envelope can be seen as approximately connecting the peaks of the fast rhythm. They also extracted dynamics for the rapidly-varying phase of the rhythm. A burst is then seen as an epoch during which the envelope exceeds some threshold. This provided insight into properties of the fluctuations of the gamma rhythm, although it did not allow the role of the noise strength to be investigated.

Here, we first directly relate the dynamics of the envelope and phase of the rhythm to all the biophysical parameters, including synaptic noise strength. From there, we develop a first passage time analysis of the envelope to quantify the mean duration of bursts as a function of the parameters. This enables us to uncover an “optimal” dynamical regime of healthy amplitude and frequency variability with similar statistics to those seen in certain *in vivo* data. Below, we use the term “amplitude” to signify the magnitude of the fast variables, as distinguished from its slowly evolving “envelope”.

For concreteness, we focus on a known simple excitatory-inhibitory recurrent network of spiking neurons with self-couplings exhibiting gamma oscillations, and investigate its ability to produce bursting behavior. We find two master parameters that govern burst statistics, giving much needed insight into burst generation and the correction of “faulty” burst statistics. Our simple model can also explain bursts observed in other frequency bands (such as beta), and thus constitutes a general framework for studying bursty brain rhythms.

We first motivate the choice of microscopic model and its associated formulation in terms of a noisy population firing rate model. We then derive (with details in the Methods) the noisy dynamics of the amplitude and phase of the rhythm, identify dynamical regimes of interest for gamma bursts, and perform first passage time (Fokker-Planck) analysis to characterize burst statistics. Comparisons of envelope-phase dynamics to full network simulations validate our approach. We then discuss how different combinations of biophysical parameters can underlie healthy and pathological rhythm variability.

## Results

Our starting point is the work of Xing *et al*.^20^. They showed that for the specific case of bursty gamma rhythms, LFP’s from macaque visual cortex are well modeled by a simplified version of the classic Wilson-Cowan (WC) rate model for reciprocally connected excitatory (E) and inhibitory (I) populations. The classic WC model (1972)^40^, which accounts for oscillations in such EI networks, includes neurons with a graded response beyond threshold. Our goal is to characterize, both theoretically and numerically, the effect of system parameters including noise on bursting. However, the noise incorporated into the WC model in Xing *et al*.^20^ is not properly scaled with system size (i.e. with the number of neurons) as in recent theoretical work. Furthermore, the firing rate-versus-input characteristic for their neurons was a step function. This strong nonlinearity amounts to a less realistic two-state (active-inactive) description of single neuron function, and impedes analytical work. We therefore wish to use an improved version of their WC model, closer to the classic one and that allows us to formulate a theory in the first place. This requires a smooth nonlinearity with properly scaled noise. Applications of our approach to other LFP data including from humans are currently being pursued and will appear elsewhere. We therefore begin here by discussing why we focus on quasi-cycles, then show network simulations with two-state neurons to set the stage for the WC model we will use. This is because the network with two-state neurons has been shown to be well approximated by the WC model with smooth nonlinearities and system-size dependent noise (Wallace *et al*.^41^). Then we proceed with analyzing the bursty rhythm properties of that model.

### Network Model for stochastic gamma-band activity

Two principal types of computational models have been proposed to explain the variability, and in particular the bursts responsible for the fast temporal decorrelation of gamma rhythms seen *in vivo*. The first proposes that broadband gamma rhythms result from synchronous chaos, a form of randomness that does not rely on noise but rather on the nonlinear properties of the network and the external stimulus. This requires multiple PING or ING (Interneuron Network Gamma) circuits in the presence of strong long-range excitatory connections^42,43^. The second type involves a single PING or ING circuit with a stable equilibrium, i.e. without noise all firing rates are constant; the operating regime must therefore be near the onset of oscillatory synchrony. The variability then results from the noise in the circuit^20,26,44^. We consider a simple model of this latter type, namely the network of stochastic spiking neurons in^41^, where noise is due to the probabilistic transitions between quiescent and active states of single neurons. This noise vanishes when the network has an infinite number of cells. Intrinsic to the network, this noise reflects the probabilistic nature of spiking, with probability proportional to neural input, which mimics the biophysical reality of spontaneous and input-driven neural activity.

**Figure 1:**
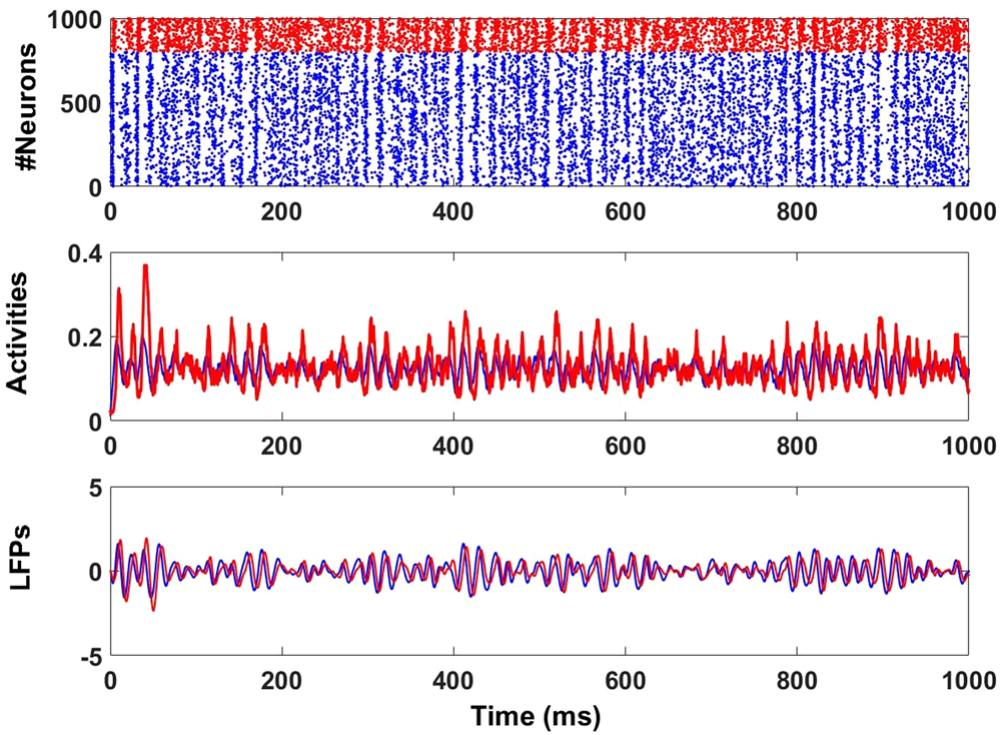
Stochastic oscillatory rhythm generated by a recurrent stochastic Wilson-Cowan (E-I) network (see Methods) working in the transient synchrony regime. Top: Raster plot. Middle: Excitatory *E*(*t*) (blue) and inhibitory *I*(*t*) (red) activities. Bottom: Excitatory (blue) and inhibitory (red) LFPs. They show epochs of high amplitude corresponding to synchronized activity followed by epochs of low amplitude corresponding to desynchronized or less synchronized activity. Excitatory and inhibitory activities and their corresponding LFPs display a slight phase difference. The raster plot and activities were simulated using the exact Gillespie algorithm^41,95^). The LFPs were obtained by first removing the signal means from the respective excitatory and inhibitory activities, followed by filtering using a Butterworth second-order filter with a lower cutoff frequency of 20 Hz and upper cutoff frequency of 100 Hz. The parameters are as in Table 1 excepted *W*_*ee*_ = 25.3.

This simple network reproduces features such as bursts of population synchrony and irregular single neuron firing as seen *in vivo* and in more realistic networks. In addition, mean-field analysis^45^ shows that such a network is a stochastic version of the well-known Wilson-Cowan firing rate model^40^. Average quantities like activities or LFPs can be described by analytical equations (see Methods), which is not generally possible for complex network models. This stochastic Wilson-Cowan model, like more complex biophysical network models^26^, exhibits two oscillatory regimes. The ***Transient synchrony*** regime is one where noise is required to see oscillations, i.e. the noise induces them. In this regime, oscillations appear during transient epochs of network synchronization called “bursts” with varying lifetimes (Fig. 1). In contrast, the ***High synchrony*** regime does not exhibit bursts for small noise; highly coherent oscillations occur in the absence of noise. The level of network synchronization is always high, and epochs of desynchronization are very rare. Moreover, the model can generate oscillations in several frequency bands (beta, gamma, and high gamma) and exhibits other non-oscillatory dynamics like the asynchronous regime. Direct simulations of the model use the exact Gillespie algorithm^46^. With parameters in the ***transient synchrony*** regime, it is possible to extract the activities of the excitatory and inhibitory populations and their corresponding LFPs (see Fig. 1, lower panel).

**Table 1:** Model parameters, definition and value.

Parameter	Desription	value
*α*_*E*_	decay rate of an excitatory cell	0.1
*α*_*I*_	decay rate of an inhibitory cell	0.2
*β*_*E*_	maximal firing rate of an excitatory cell	1
*β*_*I*_	maximal firing rate of an inhibitory cell	2
*h*_*E*_	External input to the excitatory population	−3.8
*h*_*I*_	External input to the inhibitory population	−8
*W*_*ee*_	Recurrent excitatory synaptic coefficient	27.4
*W*_*ii*_	Recurrent inhibitory synaptic coefficient	1.3
*W*_*ei*_	Synaptic connection from inhibitory to excitatory cells	26.3
*W*_*ie*_	Synaptic connection from excitatory to inhibitory cells	32
*N*_*E*_	Number of excitatory cells	800
*N*_*I*_	Number of inhibitory cells	200

Parameters values used throughout this paper, unless specified in the caption of certain figures.

### Local Field Potentials (LFPs) can be described by stochastic linear equations

Recorded activities *in vivo* are usually filtered according to the frequency band of interest before analysis. Filtered activities are then considered as a measure of LFPs^47,48^. A similar method can be applied to network activities extracted directly from numerical simulations Fig. 1(Bottom). The filtering used here first removes the mean from any time-varying activity to keep the part induced by noise; broadband gamma activity *in vivo* has in fact been likened to filtered noise^20–22^. The zero-mean activity is then filtered using a bandpass filter with lower and higher cutoff frequencies in the gamma band limits Fig. 1 (Bottom).

A similar result can be achieved analytically by deriving the dynamics of the stochastic parts of the activities. From mean field analysis^41,49,50^, the dynamics of excitatory and inhibitory activities can be obtained in terms of the stochastic Wilson-Cowan equations, in which there is one (nonlinear) equation for each of the excitatory (E) and inhibitory (I) populations (Methods). The behavior of the E population is coupled to that of the I population and vice-versa. The linear stability analysis of the noise-free analogs of these equations (i.e. of the Wilson-Cowan equations) shows that many parameters lead to a stable fixed equilibrium (as we will see below). Oscillatory regimes correspond to the parameter ranges where the corresponding eigenvalues of the system are complex conjugates. If the real part of the eigenvalues is negative, the deterministic equations have damped oscillations; the corresponding stochastic Wilson-Cowan network operates then in the ***Transient synchrony*** regime (also know as the quasi-cycle regime) where oscillations, albeit irregular ones, are sustained by noise. This regime is very popular and has already been suggested to underlie frequency-specific, hierarchical corticocortical^51,52^ and thalamocortical^53^ interactions, although with a reduced level of complexity. The analytical treatment in the present study might very well serve as a starting point to understand these large-scale interactions at a more fundamental level. If instead the real part of the eigenvalue is positive, the nonlinear deterministic equations exhibit coherent oscillations with almost constant amplitude and frequency; the stochastic network is then in the ***High synchrony*** regime where the noise has a relatively smaller effect. Mathematically, the transition from the transient to the high synchrony regime upon changing parameters corresponds to a Hopf bifurcation.

A Linear Noise Approximation (LNA) further yields a linear approximation to the stochastic nonlinear dynamics for the LFPs^41,45,49,50^:

1 $$\frac{d{\mathop{V}\limits^{ \sim }}_{E}(t)}{dt}={A}_{11}{\mathop{V}\limits^{ \sim }}_{E}(t)+{A}_{12}{\mathop{V}\limits^{ \sim }}_{I}(t)+{\sigma }_{E}{\eta }_{E}(t)$$

2 $$\frac{d{\tilde{V}}_{I}(t)}{dt}={A}_{21}{\tilde{V}}_{E}(t)+{A}_{22}{\tilde{V}}_{I}(t)+{\sigma }_{I}{\eta }_{I}(t)$$

The quantities ${\tilde{V}}_{E}$ and ${\tilde{V}}_{I}$ represent the excitatory and inhibitory LFPs respectively, and their time evolutions again depend on one another. The coefficients *A*_*i*,*j*_ (i, j = 1, 2) and the noise strengths *σ*_*E*_ and *σ*_*I*_ all depend on the single cell and connectivity parameters of the original nonlinear system (Methods). The inputs *η*_*E*_ and *η*_*I*_ are two independent zero-mean Gaussian white noises. The LFPs, which are filtered, zero-mean versions of the activities, can also be seen here as filtered versions of two white noises driving the recurrent E-I network^20^, where the filter parameters are the *A*_*i*,*j*_’s.

The amplitudes of the LFPs directly simulated from the two coupled linear stochastic equations fluctuate stochastically^38^. The same is true for the frequency which also exhibits variability in the gamma band; not surprisingly, the E and I phases are stochastic. A closer inspection reveals that the epochs of nearly constant phase correspond to epochs of high LFP amplitudes (gamma bursts)^38^. Such noisy filtered signals exhibit the stochasticity and the bursting structure of recorded LFPs *in vivo*^20–22^. Moreover, analytical studies of these signals (Methods) reveal properties such as the approximately constant ratio of LFPs from E and I cells, and approximately constant phase differences^38^.

**Figure 2:**
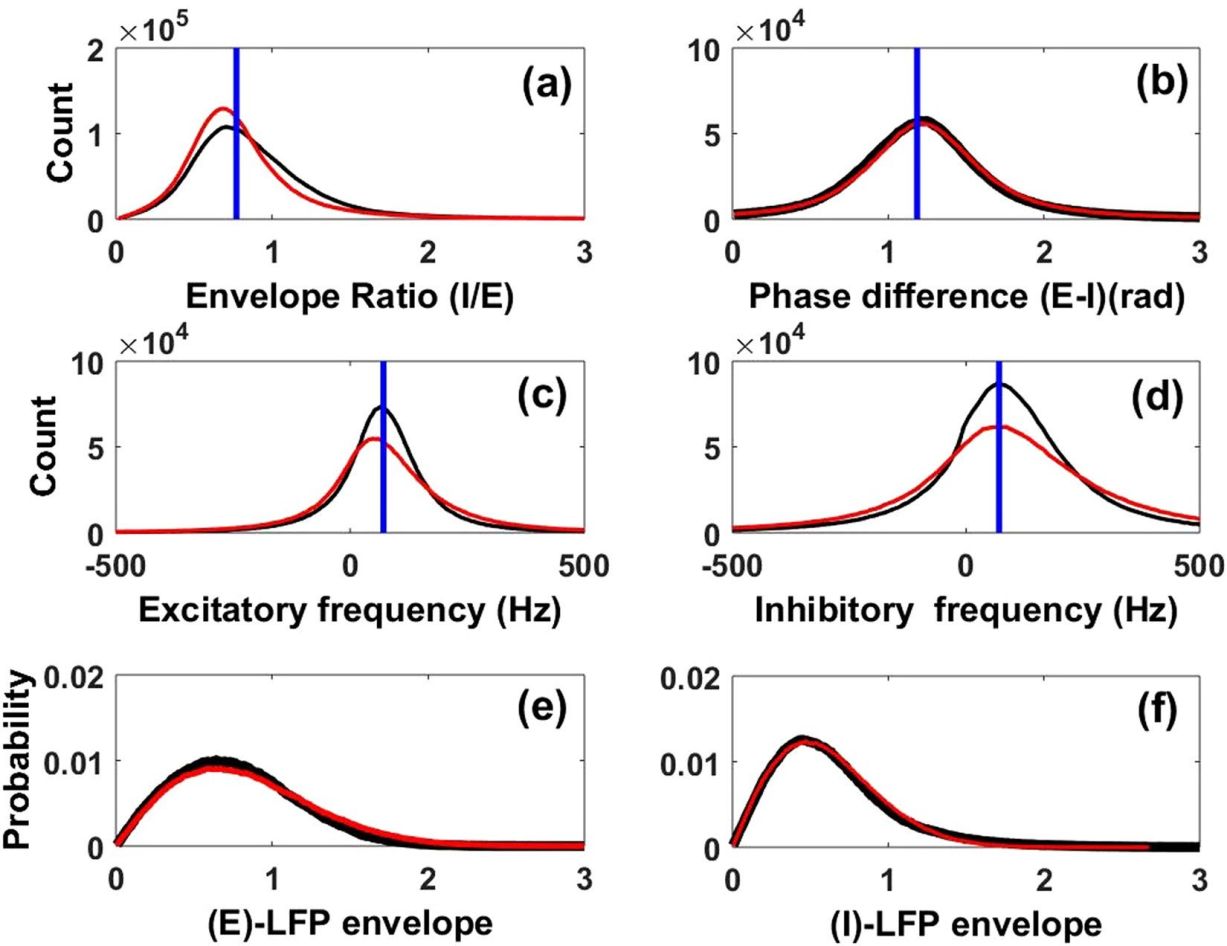
Properties of analytic versus filtered LFPs. Properties of the envelope and phase of the excitatory and inhibitory LFPs in Eqs. 1 and 2 obtained via the analytic signal technique (see Methods). Shown are the distributions of (**a**) the ratio of the envelopes of the inhibitory and excitatory LFPs (I/E), (**b**) the phase difference between E and I LFPs (**c**), the instantaneous frequency of the excitatory LFP, (**d**) the instantaneous frequency of the inhibitory LFP, (**e**) the envelope of the excitatory LFP envelope, and (**f**) the envelope of the inhibitory LFP. For all panels, distributions in black come from exact numerical simulations of the full nonlinear stochastic Wilson-Cowan neural network with 2-state neurons (Fig. 1 bottom panel), while those in red come from the approximate linear stochastic model, Eqs. 1 and 2. In panels (a–d), the vertical blue lines represent analytical predictions of the means of those distributions (Methods). For panels (a–b), the means were computed using Eq. 19, while for panels (c–d) we use the expression of *ω*_0_ right after Eq. 6. The instantaneous frequencies (Panels (e–f)) are obtained as in^38^.

The same properties are present in LFPs extracted directly from simulations of the full microscopic nonlinear network. Figure 2 presents properties of the envelopes and phases of the LFPs for this full nonlinear network, for its linear approximation using only two equations (Eqs. 1 and 2), and corresponding theoretical predictions. This serves as a guide for the modeling hypotheses we make below to derive envelope-phase dynamics. It is clear that the envelope and phase properties for the full nonlinear network are in good agreement with those obtained from simulations of the linear stochastic dynamics (Fig. 2(a,b)). Frequencies present in the LFP have a mean in the gamma band (Fig. 2(c,d)); their distribution agrees well with that of the rhythms extracted from simulations of the full nonlinear network. LFP envelope distributions from the linear and nonlinear systems are also in good agreement (Fig. 2(e,f)). Thus LFPs generated using the simple linear stochastic equations are statistically similar to those extracted from the full nonlinear excitatory-inhibitory network, which themselves are similar to recorded LFPs *in vivo*^20^. Note that instead of considering a single LFP measure, namely the sum of the excitatory and inhibitory LFPs as is often done, here for completeness the two quantities are analyzed separately; they are linked by their ratio and phase difference as shown in Fig. 2(a,b).

### Envelope and Phase equations

We consider the coupled stochastic equations for the LFP dynamics in the transient synchrony regime. The goal is to derive equations governing the time evolution of the envelopes and phases of excitatory and inhibitory LFPs described in Eqs. 1 and 2. We make three hypotheses about the LFP properties (Fig. 2):

1. The distribution of the ratio between excitatory and inhibitory LFP envelopes is approximately Gaussian^38^, as shown in Fig. 2(a). Instead, a constant ratio is assumed, whose value equals the mean of the associated Gaussian distribution. This choice is made because in the PING model the numbers of E and I cells which fired during an oscillation cycle are almost proportional. This has been observed in a computational study of a more complex network^54^ and *in vivo* as well^55^.
2. The phase difference between excitatory and inhibitory oscillations is also approximately Gaussian^38^ as observed in Fig. 2(b). A constant phase difference is assumed, and made equal to the mean of the corresponding Gaussian distribution. This choice is based on the fact that the inhibitory neurons fire a small delay time after excitatory neurons during each oscillation cycle (this delay is smaller than the period of the oscillation), a known property of the PING model (Fig. 1 bottom)^41,56^.
3. The frequencies of the excitatory and inhibitory LFPs have moderate variability but are also approximately Gaussian (Fig. 2(c,d)); we choose the mean of those distributions as the mean LFP frequencies.

Without loss of generality, we seek an expression for the excitatory LFP in a sinusoidal form, with an envelope *Z*_*E*_(*t*) and phase *ϕ*_*E*_(*t*) and a constant mean frequency *ω*_0_ to be defined below. The dynamics of the inhibitory LFP can be directly derived from this expression using the three assumptions above. The envelope ratio and phase difference between the excitatory and inhibitory LFPs are computed from the linear stochastic Eqs. 1 and 2 as

3 $$\alpha \equiv {\textstyle \langle }\frac{Env[{\mathop{V}\limits^{ \sim }}_{I}(t)]}{Env[{\mathop{V}\limits^{ \sim }}_{E}(t)]}{\textstyle \rangle }\,\,and\,\,\delta \equiv {\textstyle \langle }Arg[{\mathop{V}\limits^{ \sim }}_{E}(t)]-Arg[{\mathop{V}\limits^{ \sim }}_{I}(t)]{\textstyle \rangle }$$

where 〈.〉 can be considered a time average of the stochastic process in Eqs. 1 and 2. The envelope *Env* is defined as the magnitude of the analytic signal associated with the LFP (see Methods). Likewise, $Arg[{\tilde{V}}_{E}]$ is the phase angle of the analytic signal. We choose to work with the excitatory LFP in the form

4 $${V}_{E}(t)={Z}_{E}(t)\cos ({\omega }_{0}t+{\varphi }_{E}(t))\,.$$

We seek the functions *Z*_*E*_(*t*) and *ϕ*_*E*_(*t*) by substituting Eq. 4 into Eq. 3 to first obtain their inhibitory counterparts, then inserting both into Eqs. 1 and 2 and finally applying the *Stochastic Averaging Method* (SAM) (see Methods). This yields the following dynamics of the envelope and phase (see Eq. 35):

5 $$d{Z}_{E}(t)={\textstyle (}\,-\,\nu {Z}_{E}(t)+\frac{D}{2{Z}_{E}(t)}{\textstyle )}dt+\sqrt{D}\,d{W}_{1}(t)$$

6 $$d{\varphi }_{E}(t)=\frac{\sqrt{D}}{{Z}_{E}(t)}\,d{W}_{2}(t)$$

where

$$\nu =-\,\frac{{A}_{11}+{A}_{22}}{2},\,{\omega }_{0}=\frac{1}{2}\sqrt{-{({A}_{11}-{A}_{22})}^{2}-4{A}_{12}{A}_{21}}\,\,{\rm{a}}{\rm{n}}{\rm{d}}\,\,D=-\,\frac{{A}_{12}}{2{\omega }_{0}^{2}}{\textstyle (}\,-\,{A}_{12}{\sigma }_{I}^{2}+{A}_{21}{\sigma }_{E}^{2}{\textstyle )}.$$

Here, *W*_1_(*t*) and *W*_2_(*t*) are independent Brownian motions (their time derivatives are Gaussian white noises), ***ν*** is the absolute value of the real part of the eigenvalues of the Eqs. 1 and 2 with zero noise, and *ω*_0_ is the peak frequency. The effective noise strength *D* in the envelope equation depends on the network coefficients governing the linear stochastic dynamics of the E and I populations, in particular on the two noise intensities. In the transient synchrony regime, *D* is either positive (*A*_12_ is always negative, and *A*_21_ is always positive), or zero if both those intensities are zero.

Inspection of these dynamics reveals that the time evolutions of the envelope and the phase are both driven by noises. With *D* = 0, the envelope decays to zero, and the phase remains constant. This is again in agreement with the idea that gamma-band LFPs are close to filtered noise in this description. In particular, the envelope equation highlights the importance of noise for the appearance of bursts in the LFP dynamics.

Interestingly the dynamics of the envelope of the LFP is not coupled to that of the phase in this approximation; the reverse is not true, as the phase evolution depends on the envelope. The phase undergoes a Brownian motion with envelope-dependent intensity. In contrast with^38^, our envelope-phase decomposition refers directly to all network parameters through *ν* and *D* and shows clearly the importance of the noise for the LFP dynamics. Consequently, it is easier with our description to investigate how different network parameters effectively shape the bursting structure of LFPs. In addition, our approach does not theoretically require the limitation *ν*/*ω*_0_ ≪ 1 as in^38^. In fact, we tested our approach for values of *ν*/*ω*_0_ even close to one and found a good agreement with the corresponding Eqs. 1 and 2 (not shown).

**Figure 3:**
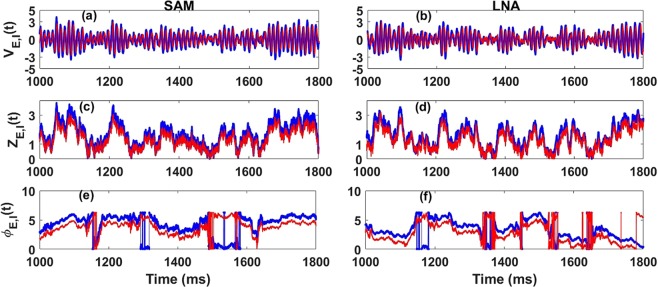
Dynamics of the LFPs, their envelopes and their phases components from Eqs. 1 and 2, Eqs. 5 and 6 and Eq. 4 (also Eq. 22 in Methods). (**b**,**d**,**f**) LFPs, envelopes and phases from the Linear Noise Approximation (LNA) Eqs. 1 and 2. (**a**,**c**,**e**) LFPs, envelopes and phases from SAM Eqs. 5 and 6 and Eq. 4 (also Eq. 22 in Methods). Like for the previous figures, blue corresponds to excitatory components and red to inhibitory ones. In the SAM case, the envelope and phase processes were simulated using two independent OU processes (see Methods, Eq. 37, Eq. 41), integrated using the Euler-Maruyama method. The envelope and phase dynamics in the LNA case were obtained by applying hilbert transform on the excitatory and inhibitory LFPs (${\tilde{V}}_{E,I}(t)$). The parameters are taken from Table 1.

Equation 5 is well-known in the statistics literature and is associated with the Rayleigh Process which describes the envelope of a periodic Gaussian process with uniformly distributed phase^57,58^. It also finds applications in the theory of stochastic mechanical and seismic vibrations where it models the envelope of a damped harmonic oscillator sustained by noise^59^. Equations 5, 6 both represent the envelope and the phase of a 2-dimensional independent Ornstein-Uhlenbeck process with parameters *ν* and *D* see^57^. The uncoupling of the envelope and phase equations allows a derivation of certain statistical properties such as the joint probability of envelope and phase^59^. The dynamics of the inhibitory LFP can be easily recovered from Eqs. 3–4 (see Eq. 22 in Methods). Numerical simulations of LFPs derived from these envelope-phase equations show similar statistical properties as the simulated LFPs from the linear model driven by additive noise in Eqs. 1 and 2 (see Fig. 3 and Methods).

From an experimental standpoint, it is of interest to know the proportion of time that the process spends near different envelope values. This can be obtained by computing the probability density for the process, either theoretically (if possible), or in an approximate form using numerical simulations of the process. The density for the envelope can in fact be computed analytically as the stationary density of the Fokker-Planck equation Eq. 41 obtained from Eq. 5, namely Eq. 42 in the Methods section:

7 $$P({Z}_{E})={\textstyle (}\frac{2\nu }{D}{\textstyle )}{Z}_{E}\exp {\textstyle (}\,-\,\frac{\nu }{D}{Z}_{E}^{2}{\textstyle )}.$$

The peak *R* of the stationary density of this noisy envelope process lies at

8 $$R\equiv \sqrt{\frac{D}{2\nu }}.$$

The peak value *R*, which is the most probable envelope amplitude value, will be used below as a measure of the degree of network synchronization. A low value of *R* reflects the fact that the network is poorly synchronized, and can’t build up a strong oscillation; conversely, a high value of *R* implies a strong degree of network synchrony leading to strong oscillations in the recurrent circuit. One could use a more standard measure of the network oscillatory strength, such as a spectral coherence measure; generally we expect such measures to be proportional to *R* in this transient synchrony regime. But we have focused instead on a measure that is directly relevant to the envelope bursts.

We have thus provided a derivation for the envelope-phase dynamics for gamma oscillations in the transient synchrony regime that explicitly includes dependencies on all the parameters of the original full nonlinear model. The envelope-phase model is able to exhibit transient oscillations - and hence bursts - in other frequency bands by changing synaptic coefficients or synaptic time constants.

### Envelope dynamics suggests distinct types of fluctuation amplification

**Figure 4:**
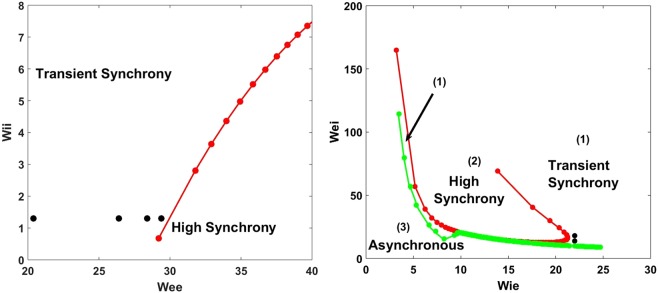
Different dynamics of the stochastic spiking network in the parameter space. Left: Recurrent plane (*W*_*ee*_,*W*_*ii*_). Black dots correspond to the four different values of the parameter *R*, obtained from left to right using the parameters (**a**) *W*_*ee*_ = 20.4, *ν* = 0.0648, *D* = 0.0512, *R* = 0.6288. (**b**) *W*_*ee*_ = 27.4, *ν* = 0.0182, *D* = 0.0613, *R* = 1.2999. (**c**) *W*_*ee*_ = 28.4, *ν* = 0.0110, *D* = 0.0613, *R* = 1.6900. (**d**) *W*_*ee*_ = 29.4, *ν* = 0.0038, *D* = 0.0648, *R* = 2.9194. Right: Feed-forward plane (*W*_*ie*_,*W*_*ei*_). Red and green curves with dots correspond respectively to the bifurcation lines between the transient and high synchrony regimes and the transient synchrony and asynchronous regimes. Left: The green bifurcation curve was plotted by setting *ν* = 0 through linear stability analysis (see Methods, Eq. 21). The transient synchrony regime then corresponds to the area *ν* < 0 and the high synchrony regime to *ν* > 0. Right: The red curve corresponds to the transition between the two oscillatory regimes as described in the left case. The green curve corresponds to the case *ω*_0_ = 0 and the asynchronous regime corresponds to the case where *ν* < 0. The two black dots in the right panel refer to two points at the same distance of the transition but at different frequencies (the diagram of frequencies is not displayed here). *W*_*ei*_ sets the strength of the feedback inhibition received by the excitatory population, and *W*_*ie*_ sets the strength of the feedback excitation received by the inhibitory population. And *W*_*ee*_ and *W*_*ii*_ are respectively the strengths recurrent excitation (excitation received by the excitatory population from itself) and recurrent inhibition (inhibition received by the inhibitory population from itself). For this right panel we have chosen *h*_*E*_ = −7 instead of *h*_*E*_ = −8 as in all other figures.

Our envelope-phase equations depend on network parameters through *ν* and *D* which are functions of all the parameters of the original network model of the LFPs. We first investigate how these parameters lead to different network dynamics. We aim to understand this dependence in terms of connectivity parameters by varying two of them at a time while keeping constant the two others as well as all other parameters. In the plane of the parameters governing the strength of recurrent connections, (*W*_*ee*_, *W*_*ii*_), we identify two easily separable regimes: transient synchrony and high synchrony (Fig. 4 left panel). The plane of the parameters governing feedforward connectivity, (*W*_*ei*_, *W*_*ie*_), gives a more complex array of possible transitions, and involves a third regime: an asynchronous non-oscillatory state. Two types of transitions from transient synchrony can occur: one to a high synchrony regime via a Hopf bifurcation (an equilibrium gives way to a periodic activity pattern), and another to the asynchronous regime (Fig. 4 right panel).

The value of *R* controls the magnitude of the envelope fluctuations, which in turn reflect different degrees of amplification of the white noise fluctuations that drive the E-I system. It also reflects the competition between the internal network noise and the deterministic oscillation. We can increase *R* by either decreasing the value of *ν* (which depends on *A*_11_ and *A*_22_) at constant effective noise strength *D*, or increase the value of *D* at constant *ν*, or increase *D* while decreasing *ν*.

In the first scenario, decreasing *ν* increases the damping time of oscillations, i.e. they are longer-lived. This scenario affects both the amplification of the fluctuations, i.e. the burst size, as well as the duration of these amplifications, i.e. the burst duration; without fluctuations, the rhythm would just die out. Such envelope amplification has been observed both in computational studies and *in vivo* in another frequency band^54,60^. A simple way to implement this scenario in our model is to increase the recurrent excitation. This reduces the value of *ν* without significantly changing *D*; this can be seen from the fact that *ν* clearly depends on *W*_*ee*_, and *D* depends on *W*_*ee*_ through *ω*_0_ (see expressions below Eq. 6 and in Methods).

The second scenario corresponds to a different type of amplification since it does not change *ν*; it thus keeps the amplification duration constant. We do not detail this type of amplification; its complexity requires an elaborate treatment that goes beyond our study. We verified that feedback inhibition can cause the increase of D at constant *ν* (we do not detail it here; however the expression of *D* depends clearly on *W*_*ei*_ through *A*_12_, see below Eq. 6 and Methods). The third scenario is a mixture of the previous two.

**Figure 5:**
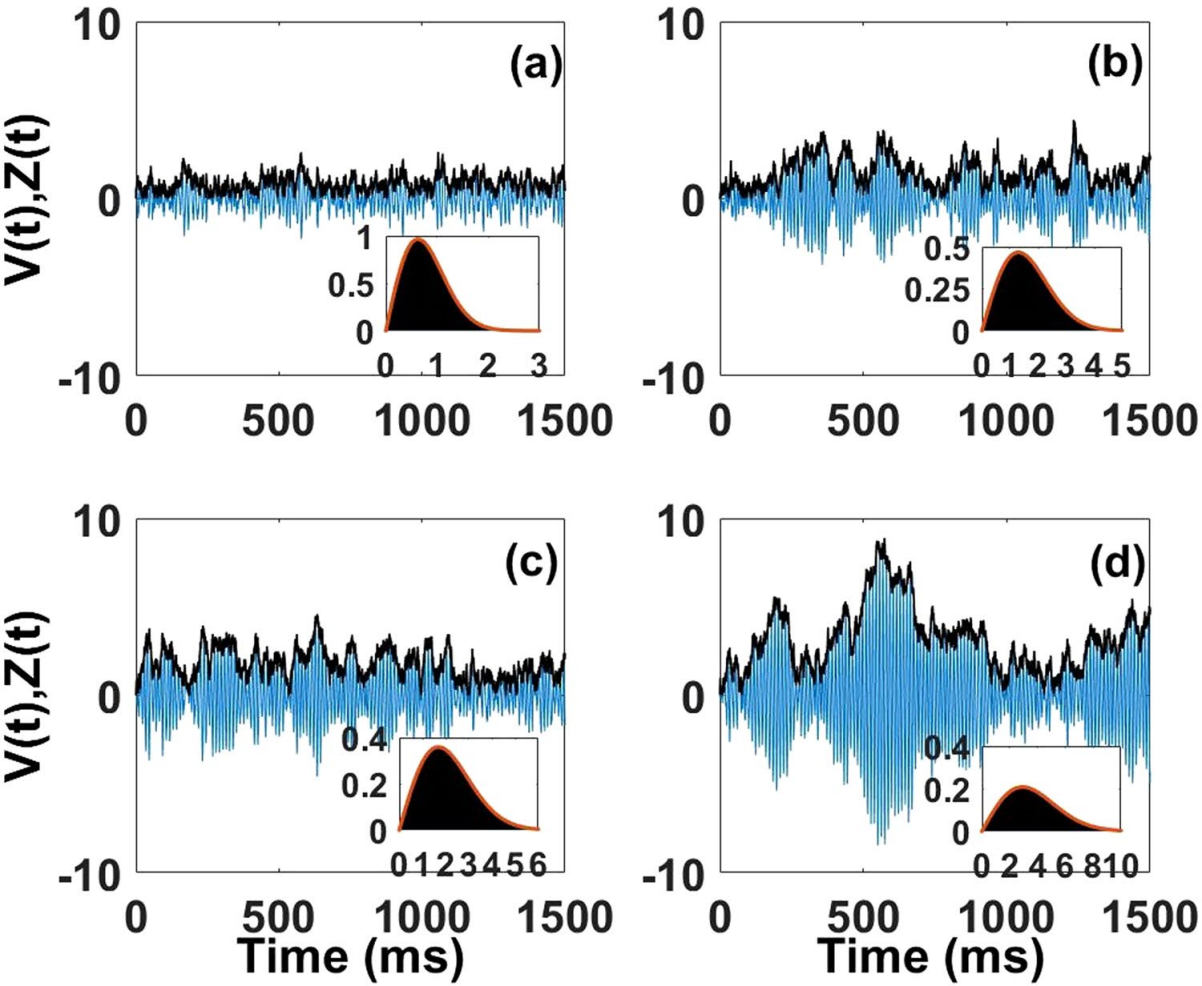
Dynamics of the envelope fluctuations (black) and their associated LFPs (blue) for the same four parameter values used in Fig. 4: (**a**) R = 0.6288, (**b**) R = 1.2999, (**c**) R = 1.6900, and (**d**) R = 2.9194. Insets show the corresponding probability densities for these fluctuations, computed both numerically as well as analytically using Eq. 42 (red curve). Note how the size of the fluctuations and their durations increase as *R* increases, i.e. as the network becomes more synchronized.

The first scenario is appealing for our purposes since it yields rhythms similar to those seen in healthy and diseased states. We consider four points along a horizontal line in the recurrent plane of parameter space (Fig. 4 left panel), lying increasingly closer to the transition between the transient and the high synchrony regimes. As *R* increases, so does the network synchronization (Fig. 5), although the peak frequency of the rhythm stays around 85 Hz for our choice of parameters. Far from the transition, i.e. for a low value of *R*, there is a lack of synchronization (Fig. 5(a)). The density of the envelope values has low variance (Fig. 5(a) inset). But the mode of this density and the duration of bursts are likely too small to support reliable communication through coherent oscillations. The notion of burst itself is compromised as it is difficult to extract from the surrounding small fluctuations. In addition, a similar lack of synchronization is observed in patients suffering of schizophrenia (negative symptoms)^61–63^ and constitutes one of the common markers of this pathology.

A working point too close to the transition, corresponding to a high value of *R*, leads to strong synchronization (Fig. 5(d)). The broadness of the envelope distribution means high variability of the underlying amplitude value (Fig. 5(inset)). Burst durations can last more than 1 second. However, it has been argued that such excessive synchronization could lead to the repetition of the same message and impede the transmission of other messages^64^. This could also destroy the flexible routing of information observed when synchronization is more moderate^26^. Also, such long burst durations go counter to the fast temporal decorrelation of gamma band activity observed *in vivo*^65,66^. They have been associated with dysfunctions resulting from an excess of excitation or lack of inhibition which lead to sustained high envelope amplitude as seen in epilepsy^67–69^ or Attention Deficit Hyperactivity Disorder (ADHD)^70^.

Between these extremes, we show two working points with intermediate values of *R* (Fig. 5(b,c)). There we find moderate envelope values and burst durations (Fig. 5(b,c)). This suggests an optimal brain state between excess and lack of synchronization. Here and below, we use the word “optimal” to describe a range of parameters, rather than a specific set of parameter values, for which the burst statistics resemble those seen in healthy recordings from the monkeys. We can in fact propose three regimes in the transient synchrony regime: a **noise-dominated** regime at low *R*, an **oscillation-dominated** regime at high *R*, and an **oscillation-noise** regime at intermediate values of *R*. We can then assign pathologies related to lack of synchronization to the **noise-dominated** regime, those related to excessive synchronization to the **oscillation-dominated** regime, and healthy states to the **oscillation-noise** regime. The fact that the **oscillation-noise** regime covers a range of parameters relates to the fact that different healthy subjects can exhibit different gamma amplitude modulations.

Along a vertical line in the (*W*_*ie*_, *W*_*ei*_) parameter space (Fig. 4 right), two points at a relative same distance to the transition line lead to rhythms that can differ significantly in their peak frequency (not shown). The amplitude modulations are however similar. Such points could correspond to separate brain states, such as awake or anesthetized, as reported in^20^. Our envelope-phase equations provide a simple explanation of how, in biophysical terms, different amplitude modulations of brain rhythms can relate to different brain states, assuming basic E-I connectivity.

### Dynamics and statistics of Gamma bursts

#### Burst extraction

We define gamma bursts formally as epochs where the envelope process is sustained above a specific threshold. The corresponding burst duration is, therefore, the time the process spends above that threshold. Burst durations recorded *in vivo* have short mean values (on the order 100 ms). Our envelope process is not coupled with the phase process and allows in principle the derivation of the mean burst duration in terms of mean first passage times (MFPT) away from the threshold and back to it. Our derivation (see Eq. 51 in Methods) gives the following approximate mean burst duration in terms of network parameters:

$$T={\textstyle (}\frac{1}{2\nu }{\textstyle )}{\textstyle [}\exp {\textstyle (}\,-\,\frac{1}{2}{{\textstyle (}\frac{b}{R}{\textstyle )}}^{2}{\textstyle )}-\exp {\textstyle (}\,-\,\frac{1}{2}{{\textstyle (}\frac{c}{R}{\textstyle )}}^{2}{\textstyle )}{\textstyle ]}{\textstyle [}Ei{\textstyle (}\,-\,\frac{1}{2}{{\textstyle (}\frac{c}{R}{\textstyle )}}^{2}{\textstyle )}-Ei{\textstyle (}\,-\,\frac{1}{2}{{\textstyle (}\frac{b}{R}{\textstyle )}}^{2}{\textstyle )}{\textstyle ]},$$

with *b* ≈0.59*R* and

$$c=R{\textstyle (}\sqrt{\frac{\pi }{2}}+\sqrt{\frac{4-\pi }{2}}{\textstyle )}.$$

Here *Ei* is the exponential integral function, *b* is the threshold and *c* is an estimate of a typical maximal value that the process can reach during the burst.

Choosing the values of *b* and *c* to reveal burst characteristics regardless of the magnitude and duration scales of the fluctuations, as we did here, requires that these values be proportional to *R*. This enables the extraction of bursts and estimates of burst duration using a threshold and maximum value that scale with the mean size of the envelope fluctuations. Other choices are possible, but with this choice, the substantial variation of the burst duration is governed by the value of ν only (in the first scenario discussed at the end of the previous section). This choice would also work in the second scenario where *D* increases while *ν* is kept fixed, and the scenario where both covary.

Numerically, choosing a threshold for burst extraction is known as the *P*_*episode*_ technique^48,71–73^. This technique has the advantage that it easily detects bursts. However, it also has some limitations. The first limitation is the fact that the choice of the threshold quantitatively affects the results. The second limitation comes from the fact that, since the envelope process is a noisy signal, rapid fluctuations regularly occur that spend too little time above threshold to be considered as meaningful bursts. Such rapid fluctuations create false bursts and their consideration leads to biased exponential distributions for burst durations. To address the first point, we avoid choosing a value of threshold that is too small relative to the typical size of fluctuations, thereby averting most false bursts, or that is so high that several relevant bursts are excluded.

After choosing the threshold *b*, we deal with the second limitation by implementing a second “threshold”, or rather, second criterion: a fluctuation is considered a burst only if its envelope exceeds the mean of the envelope process for at least two oscillation cycles. This removes the artefactual short bursts, keeping only proper bursts. Further testing has revealed that changing the threshold value only modifies our results quantitatively rather than qualitatively. For example, increasing (decreasing) *b* slightly decreases (increases) the mode of the density of burst durations.

Gamma burst extraction in previous studies has been done using time-frequency analysis of the LFP. This involves thresholding the power of the smoothed version of the LFP. The advantage of those analyses is that they return both the burst duration and peak frequency content of the LFP. This usually allows one to compute marginal distributions of burst duration and burst peak frequency. From those distributions, one can calculate the mean burst duration and the mean burst peak frequency. Here, burst extraction using our criteria naturally returns a range of durations. Furthermore, to obtain the range of associated peak frequencies, we computed the corresponding peak frequency in each burst using the corresponding LFP epoch. With the set of burst durations and their corresponding peak frequencies, we can then compute the marginal distributions of the burst duration, their corresponding peak frequency, and their associated mean values.

#### Burst duration and peak frequency: mean and variability

**Figure 6:**
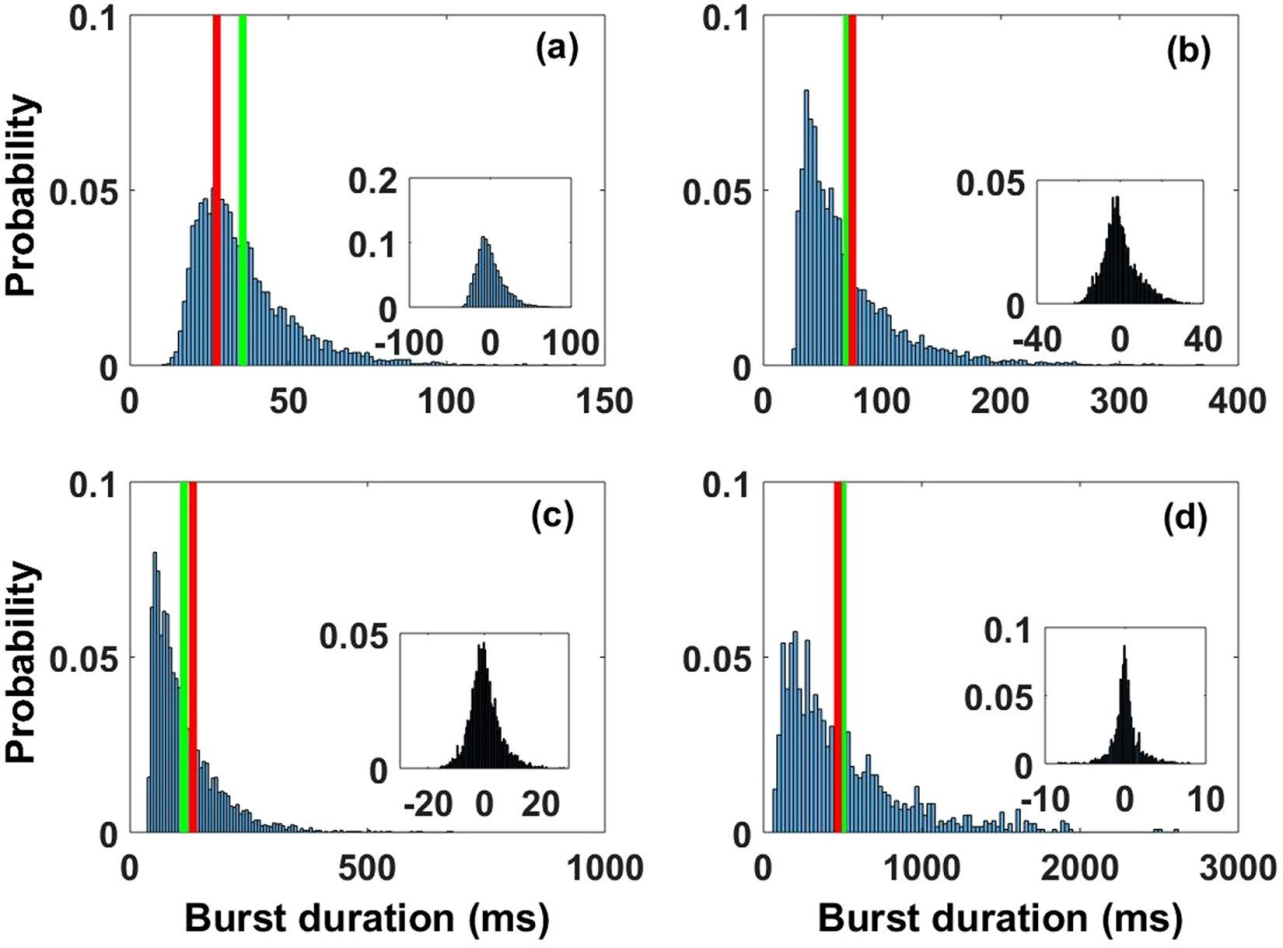
Distributions of burst durations (histograms in blue), their corresponding means (vertical line in green) and theoretical mean values (vertical line red). Distributions in insets correspond to associated peak frequency variability in Hz. Theoretical values were computed from Eq. 51 and the value of *c* was chosen as the sum of the mean and the standard deviation of each envelope process (Methods). The four cases correspond respectively to different values of *R* in the previous figures, namely (**a**) R = 0.6288, (**b**) R = 1.2999, (**c**) R = 1.6900, and (**d**) R = 2.9194. The mean burst duration increases as we get closer to the transition between the High and transient synchrony regimes and the corresponding peak frequency variability decreases. The mean values computed from histograms (vertical green lines) are, respectively, 35.00 ms, 74.50 ms, 112.25 ms and 514.60 ms, and those from Eq. 51 (vertical red lines) are 27 ms, 86.10 ms, 132.90 ms and 465.50 ms. The associated standard deviations of the peak frequency variability are respectively SD1 = 19.1 Hz, SD2 = 8.1 Hz, SD3 = 5.4 Hz and SD4 = 1.6 Hz. Furthermore, we observe mean burst durations with corresponding peak frequency variability in the range of the experimental observation for the two intermediate working points (**b**) and (**c**).

The mean burst duration observed *in vivo* is usually short (less than 100 ms on average) but its exact value varies depending on the brain state and animal subject, as well as the accuracy of the method used to extract bursts^20,21^. A normal mean burst duration observed in data is in the range (60–150 ms)^20,21^. The mean burst durations computed from marginal distributions and from Eq. 51 vary across parameter space, and thus for the four *R* values of interest in our study (Fig. 6). We observe an increase in the mean values as the transition between the transient and high synchrony regimes is approached (Fig. 4 and insets). The precise values are respectively 35.00 ms (a), 74.50 ms (b), 112.25 ms (c) and 514.60 ms (d) for the four points in the phase parameters (see green and red vertical bars in Fig. 6, computed from burst duration marginal distributions and from Eq. 51, respectively). The durations computed in (b) and (c) fall inside the *in vivo* range. The duration of 35.00 ms calculated in (a) is too short and the corresponding envelope amplification too weak compared to *in vivo* recordings. Such short durations are not seen in healthy subjects, but have been seen in psychiatric disorders such as schizophrenia. Further, the duration of 514.60 ms observed in (d) is too long, with a corresponding excessive envelope amplification uncharacteristic of healthy subjects.

Burst peak frequencies obtained in our analysis are characterized by their marginal distributions (not shown here). Unlike the burst duration distributions, the burst peak frequency distributions are approximately Gaussian. Also unlike the burst durations, the mean extracted from burst peak frequency distributions is roughly the same across the four cases (it is around 85 Hz). However, visual inspection suggests that burst peak frequency variability is reduced as the transition from transient to high synchrony is approached; this is confirmed by their distributions (Fig. 6 insets). Indeed, burst peak frequency variability is an essential marker of gamma bursts in data. We define variability (peak-frequency deviation) as the difference between absolute peak frequency for the gamma burst and the mean peak gamma frequency, averaged across all of the gamma bursts^20^. We then numerically compute from long time series a distribution of peak-frequency deviation values, and quantify the spread of this distribution by a standard deviation. This latter deviation is thus a measure of the burst peak frequency variability. We computed the standard deviations for each of the four points in the space parameter. Their values decrease as we get closer to the transition between the two regimes. The exact values of these standard deviations are respectively SD1 = 19.1 Hz (a), SD2 = 8.1 Hz (b), SD3 = 5.4 Hz (c) and SD4 = 1.6 Hz (d). We compared these values with those observed in recorded data and found that the cases (b) and (c) gave relatively good agreement.

For illustration, mean burst duration and burst peak frequency variability measures computed from recording on an anesthetized monkey in^20^ are respectively 65 ms and SD = 8.8 Hz. These values are relatively close to our case in (b) where we have a mean burst duration and a burst peak frequency variability of 74.50 ms and SD = 8.1 Hz respectively. Furthermore, data from awake and anesthetized monkeys suggests that variability decreases as mean burst duration increases. This is illustrated by a slight decrease in the burst peak frequency variability from SD = 9 Hz to SD = 8.8 Hz, following a small increase of the mean burst duration from 62 ms to 65 ms for an anesthetized and an awake monkey, respectively. This supports the relative weakness of our computed burst peak frequency variability (SD = 5.4 ms) associated with a relatively high value of the mean burst duration (112.25 ms) in case (c) of our analysis. The burst peak frequency variability in cases (b) and (c) is therefore more likely to be observed *in vivo*. For the case (a) the burst peak frequency variability SD = 19.1 Hz is too high. In contrast, the case (d) shows a reduced variability SD = 1.6 Hz, close to a highly coherent oscillation process. Such regularity disagrees with the stochastic nature of gamma-band oscillations observed *in vivo*^21^.

#### Joint distribution of burst duration and peak frequency

**Figure 7:**
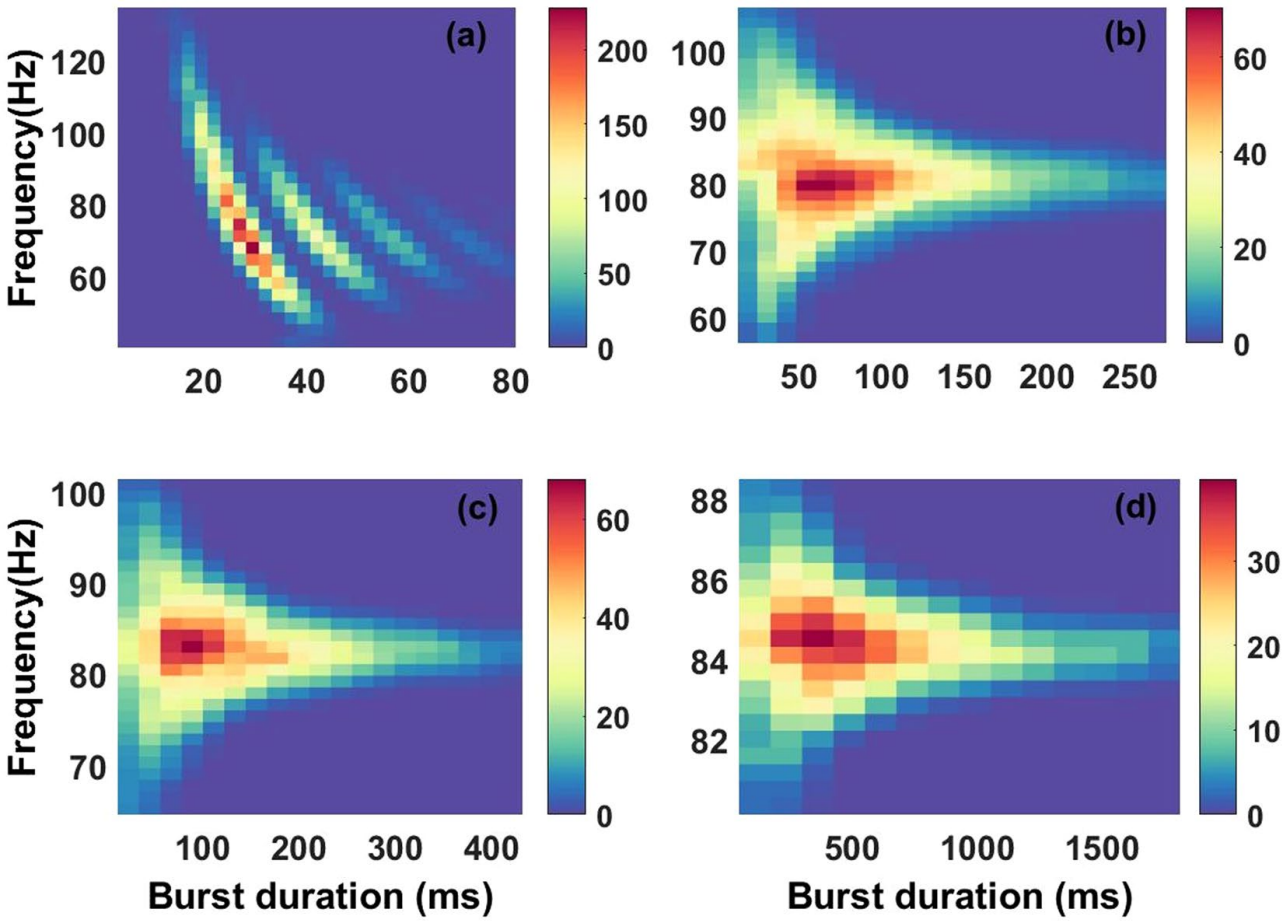
Joint distribution of burst durations and burst peak frequencies. The four cases correspond respectively to the four different values of *R* used in previous figures, namely (**a**) R = 0.6288, (**b**) R = 1.2999, (**c**) R = 1.6900, and (**d**) R = 2.9194. Panels (b,c) best represent the combinations of frequencies and burst durations seen experimentally.

Next, we investigate the count of occurrences of a burst at a given oscillatory frequency with a specific duration. This is done using the joint distribution of burst duration and peak frequency^20,21^. Such distributions are investigated over the same four values of *R* (Fig. 7). The first case (Fig. 7(a)) does not show any structure close to what is observed in the data, as the bursts are too short and frequencies quite high. In Fig. 7(d) the joint distribution shows a mode corresponding to the mean burst duration of 514 ms and peak frequency around 85 Hz. However, the lack of variability of the burst peak frequency and the excessive burst durations associated with this process disqualifies it as a model of healthy stochastic gamma oscillations observed *in vivo*.

The remaining cases (Fig. 7(b,c)) are good approximations of observed gamma oscillations^20,21^. They show modes corresponding respectively to mean burst duration and peak frequency similar to what has been done in previous computational and experimental studies^20,21,26^. We therefore conclude that there exists an optimal parameter range which reproduces the burst durations and their corresponding peak frequency variability observed *in vivo*. This region coincides with the **oscillation-noise** regime defined previously. This suggests that a mixture of intrinsic network noise and noise-free fixed point dynamics are needed to produce observed gamma oscillations. Indeed the two other regimes (noise-dominated and oscillation-dominated) both fail to reproduce *in vivo* data.

The theoretical expression of the mean burst duration Eq. 51, through its direct dependence on *R* (note the prefactor 1/*ν*) can partially explain these normal, perhaps optimal brain states. In fact, we remark that choosing an optimal state in our model corresponds first to choosing *ν* such that its inverse falls inside or is near to the range (60–150 ms) of mean burst durations seen *in vivo*. Then, we need to make sure that the value of *D* is sufficient such that the value of the amplification strength *R* is high enough (and *D* needs to be not too small relative to *ν*, because values of *D* close to zero decrease *R* to zero). In our illustration, values of (1/*ν*) for the four increasing values of *R* are, respectively, 15.43 ms, 54.9 ms, 90.90 ms and 263.15 ms, and values of *D* are almost constant around 0.06, but not too small relative to values of *ν*. The values of (1/*ν*) in (a) and (d) are clearly away from the considered range. Also the value of (1/*ν*) of the paper of^20^ is 66.67 ms and falls inside the normal range found here.

## Discussion

We obtained an envelope-phase representation of broadband gamma oscillatory LFP’s seen *in vivo*, and consequently of noisy rhythms in general, by considering a simple neural network in the PING scenario with the essential properties of excitatory and inhibitory cell types. From numerical simulations of the excitatory and inhibitory LFP dynamics, we observed that their ratio of envelopes, their phase difference as well as their respective peak frequencies all follow approximately Gaussian distributions. This allowed us to link these LFPs together, and to consider just the excitatory LFP as the network LFP. We further applied the Stochastic Averaging Method (SAM) to extract evolution equations for the slow envelope of the LFP amplitude and the corresponding phase of the LFP in terms of the parameters of the original microscopic network. The distribution of frequencies in the LFP could also be derived from the phase dynamics. The envelope-phase equations depend on all single-neuron and network parameters, and are in agreement with these quantities extracted through the analytic signal technique based on the Hilbert transform of the LFP time series.

Under certain conditions, the envelope-phase equations produce dynamics that resemble recorded LFPs *in vivo*. The model therefore provides an appropriate theoretical framework for studying LFPs of rhythms and for our ultimate goal of characterizing burst dynamics in terms of all network parameters. We have followed our formulation for that latter purpose. While many parameters govern the E-I dynamics, surprisingly few combinations of those parameters actually determine the envelope and phase dynamics. We investigated how the envelope process evolves across the parameter subspace relating to connectivity. Specifically, we chose four points in that subspace below the bifurcation between the transient synchrony and high synchrony regimes, which appears most relevant to gamma bursts. We found that the amplification of noisy perturbations - seen in large excursions of the envelope, i.e. bursts - and the corresponding burst durations increase as the transition is approached.

In the close vicinity of the transition, envelope amplifications and their durations become excessive, with possible relevance to disorders such as epilepsy and ADHD^74^. Far away from the transition, the process appears more noise-like, with the envelope exhibiting weak amplifications with very short lifetimes. The lack of synchronization in this latter case is accompanied by a reduced spectral power at gamma frequencies, and is sometimes observed in patients with neurological disorders like schizophrenia^74^. Between these two points, the two other parameter sets yield moderate amplifications and durations. These provide a better match to modulations observed *in vivo*. This suggests that there is an optimal region in the parameter space where healthy dynamics lie.

### Non-normal amplification as a mechanism for Gamma bursts

We showed that burst generation can depend on *ν* by changing *W*_*ee*_, and on *D* by changing *W*_*ei*_. The notion of an optimal region for *in vivo* gamma bursts first requires that the inverse of *ν* falls inside or lies near the healthy range (60–150 ms). But this is not sufficient, since a value of *D* close to zero will lead to a value of *R* close to zero and therefore to very little amplification; decreasing *ν* further to recover some amplification then leads to burst durations outside the healthy range. Thus, the value of *D* also has a great importance for burst generation. The parameters *ν* and *D* appear to influence distinct types of amplification, but what types specifically?

While a full answer to this question exceeds the scope of our paper, we remark that amplification in the envelope process obtained by approaching the transition (through decreasing |*ν*| in our “first scenario”) ressembles what is known as “normal amplification”. Normal amplification results from the real part of the eigenvalues of the linear noise-free dynamics being small. Very close to the transition, the absolute value of the real part of the eigenvalues, i.e. |*ν*|, approaches zero. Consequently, the amplification scales as |*ν*|^−1/2^ (Eq. 8), while the amplification duration is proportional to |*ν*|^−1^ (Eq. 51). Therefore, bursts occur with explosive amplification and very long duration. Such amplification in a neural network is mostly induced by mutual excitation among neurons, resulting from strong recurrent excitation coefficients (Fig. 4); recall that increasing *W*_*ee*_ makes *ν* tend toward zero, since *A*_11_, which increases with *W*_*ee*_, is positive and *A*_22_ < 0. In contrast, far from the transition, |*ν*| is not that small, and as a consequence, the corresponding normal amplification and its lifetime are smaller.

The two points in the middle correspond to sufficient normal amplification (not too weak and not too strong). This suggests that strong normal amplification does not agree with *in vivo* data. Furthermore, the value of *D* must be sufficient to avoid very weak amplification.

Interestingly, the amplification seen by increasing *D* at fixed *ν* (second scenario) may produce bursts that are compatible with those seen experimentally, as long as the values of *ν* are in the middle range mentioned above. Increasing *D* under these conditions has the advantage of increasing the burst magnitude without increasing burst duration. This corresponds better to the so-called non-normal amplification^75–78^. Such amplification is believed to play an important role in selective amplification observed in cat primary visual cortex (V1)^79,80^. It is also called balanced amplification because it is associated with the stabilization of strong recurrent excitation by feedback inhibition^80^. This could be the dominant amplification used by a healthy brain to produce bursts in gamma and perhaps also beta rhythms, as long as *ν* is properly set to produce normal amplification. Therefore, the two types of amplifications may underlie healthy conditions.

### Envelope-phase decomposition of more complex neural networks using SAM

Our study uses a network which does not model neurons with intrinsic voltage dynamics, and neglects the additional excitatory (AMPA and NMDA) and inhibitory (GABA-A) synaptic receptor dynamics^81^. Furthermore, noise is an intrinsic property in our network due to finite-size effects. But noise in real neural networks *in vivo* comes also from the constant bombardment of synaptic inputs, including those whose origin is outside the network^81^. Our approach could be applied to such detailed spiking networks given the approximate linear dynamics that have been extracted for those networks. For example, the Dynamic Mean Field (DMF) technique can be applied to more detailed realistic models^82–84^. DMF adequately approximates the temporal dynamics of the complex network by stochastic nonlinear equations close to our stochastic equations for the excitatory and inhibitory activities (Methods) (Eqs. 11 and 12). Such stochastic nonlinear equations can be further linearized around the stable fixed point; the resulting linear stochastic equations sustained by noise provide a fairly good approximation of the complex network dynamics^83,84^. Therefore for the purpose of studying gamma bursts in such realistic networks, it could suffice to tune parameters in the vicinity of the transient and high synchrony regimes, as in our study. The same can be said of neural field theoretic approaches with intrinsic noise to describe rhythms where linearization can be used to investigate spectra and the emergence of rhythms^85^.

Rate dynamics can also be derived for conductance-based spiking networks^86^. Such dynamics can be linearized, and our envelope-phase decomposition can be fully applied. In fact, this is true for any spiking network which can be described by 2D rate equations or 2D activity dynamics. Our approach could further be extended to networks with complex topology, such as the 2D plane model of the primary visual cortex^86,87^, or to multiple coupled E-I networks^83,84,88^. The resulting dynamics can be used to study the effect of the feedback from extrastriate cortex on visual cortex^86,87^, phase-synchronization between brain rhythms^25,27^, inter-areal brain communication^12,13,26^, functional connectivity^88–90^ or cross-frequency coupling more generally.

The extension of our model to beta rhythms may involve considering other mechanistic origins of the oscillations, such as thalamocortical loops. Likewise, bursty gamma rhythms may arise from inputs from extrastriate cortex^86^. Our method could be applied to the putative circuitry as long as the associated loop causes a damped oscillation.

It may be that bursts in one frequency range are the result of a cross-frequency interaction, i.e. between a fast rhythm and another slower rhythm both emerging from the feedback structure. Our modeling framework could still be used if the dynamics of the corresponding two networks are damped, i.e. with linearized dynamics having eigenvalues with negative real part and imaginary values corresponding to the two frequencies present.

Alternately, we could further develop our framework to describe the potential situation where a quasi-cycle in e.g. the gamma range is driven by a slower (e.g. beta) rhythm arising outside of the feedback loop. This would likely lead to stochastic amplitude-phase equations as we have described in our work, with the noise-induced rhythm being modulated by the time-dependent forcing. The mean frequency of the quasi-cycle would have to be significantly faster than the external forcing. Its mean amplitude would also have to be smaller than that of the quasi-cycle for the analysis to carry through to this driven case–otherwise, the external modulation could drag the fast rhythm in and out of the quasi-cycle regime. This analysis could be further developed to account for the transients that occur when this external input is switched on.

Preliminary simulations of a periodically forced gamma quasi-cycle reveal that the properties of the bursts change according to the frequency and the amplitude of the external input (not shown). The effect also depends on whether the forcing is applied to the excitatory population only, the inhibitory population only, or to both populations. The precise dependence of gamma burst properties on such external input parameters and network regimes is not a trivial problem, and our work in this direction promises to be a stand-alone hefty story.

### Envelope-phase decomposition of an all-to-all delayed inhibitory network

We have also investigated broadband rhythms generated by a population of stochastic two-state neurons (as in Fig. 1) but with all-to-all delayed inhibitory coupling. The delay and the proximity of a Hopf bifurcation are necessary for the appearance of the quasi-cycles^44,91^, and differs from the ING mechanism. The same transition between transient synchrony and high synchrony occurs in that model as it does in our study based on the PING mechanism. We have verified numerically, using the Hilbert transform to extract the envelope of the rhythm, that a qualitatively similar behaviour of the burst magnitude and duration occurs in this inhibitory system as the transition is approached from the transient synchrony side (not shown). We also see an analogous optimal region in the subspace of parameter space spanned by the delay and the inhibitory coupling strength, where the variability in the burst duration and in the peak frequency during bursts resembles those seen *in vivo*. This further supports the generality of our result, in the sense that the essential determinants of the burst statistics are there again the presence of noise in the vicinity of a bifurcation to synchrony. Again, to understand rhythm bursts, our envelope approach could be applied to the linearizable formalisms that have been proposed for noise-induced rhythms and their spectra in this case such as^44,91^, and^92^ in the spatio-temporal noise-driven neural field case.

Future work should also consider the statistics of bursts in the chaotic networks with long range excitatory connections that produce fast decorrelating gamma rhythms^42,43^, to see whether they exhibit qualitatively different features than those discussed here. And while our approach can easily be adapted to rhythms in other frequency bands, it does rely on linearization, and thus may not provide adequate descriptions of envelope and phase dynamics for all nonlinearities that underlie brain rhythms. One expects in those cases as well that our approach will provide a good first understanding of the parameter range underlying observed burst statistics, and what to do in case these statistics fall out of the healthy range.

## Methods

### The Model

We begin by summarizing a recent model of noisy gamma activity that is based on a network of nonlinear neurons that spike probabilistically^41^. This more biophysically realistic model is used here to illustrate gamma bursts. We then review the relation of this model to the stochastic Wilson-Cowan firing rate model and show its ability to also generate gamma bursts in terms of firing rate rather than spike events. Our envelope-phase reduction will be derived from this rate model.

The network is composed of fully connected *N*_*E*_ excitatory neurons and *N*_*I*_ inhibitory neurons. Each neuron can exist in one of the two following states: an active state (*a*) representing the firing of an action potential and its accompanying refractory period, and a quiescent state (*q*) associated with a neuron at rest. Each neuron follows a two-state Markov process. The dynamics of a neuron are specified by the transition rates between the two states. The transition probability for the *i*^*th*^ neuron to decay from the active to the quiescent state is

$${P}_{i}(active\to quiescent,in\,time\,dt)={\alpha }_{i}dt$$

where *α*_*i*_, *i* = *E*, *I* is a constant; thus this transition probability does not depend on the input to the neuron. It is typically high to mimic the largely deterministic nature of voltage reset after a spike. In contrast, the transition probability from quiescent to active is:

$${P}_{i}(quiescent\to active,in\,time\,dt)={\beta }_{i}f({s}_{i}(t))dt$$

with input

$${s}_{i}(t)=\sum _{j}{W}_{ij}{a}_{j}(t)+{h}_{i}.$$

Here *f* is the neuron input-output response function, typically a sigmoid, *W*_*ij*_ is the strength of the synaptic weight from a j-type cell onto an i-type cell (defined positive), *h*_*i*_ the external input, $\sum _{j}\,{W}_{ij}{a}_{j}(t)$ the network input and *s*_*i*_(*t*) the total input to neuron *i*. We set *a*_*i*_(*t*) = 0 if neuron *i* is quiescent and *a*_*i*_(*t*) = 1 if it is active.

At the network level, we assume that the total synaptic weight from the excitatory population to itself is *W*_*ee*_; the mean synaptic weight from an excitatory cell to another excitatory cell in the excitatory population is just *W*_*ee*_/*N*_*E*_. Similar assumptions hold for the other connection strengths, namely −*W*_*ii*_/*N*_*I*_ between inhibitory neurons, *W*_*ie*_/*N*_*e*_ from excitatory to inhibitory neurons, and −*W*_*ei*_/*N*_*I*_ from inhibitory to excitatory neurons. Also, each excitatory neuron receives the same external input *h*_*E*_; likewise, all inhibitory neurons receive the external input *h*_*I*_. The total input current *s*_*E*_ to excitatory neurons and *s*_*I*_ to inhibitory neurons are then given by

9 $${s}_{E}(t)=\frac{{W}_{ee}}{{N}_{E}}k(t)-\frac{{W}_{ei}}{{N}_{I}}l(t)+{h}_{E}$$

10 $${s}_{I}(t)=\frac{{W}_{ie}}{{N}_{E}}k(t)-\frac{{W}_{ii}}{{N}_{I}}l(t)+{h}_{I}$$

where *k*(*t*) is the number of active excitatory neurons and *l*(*t*) the number of active inhibitory neurons. This network is simulated in discrete time using the Gillespie algorithm as in^41^. A typical simulation result is shown in Fig. 1 where, in spite of the presence of a noisy rhythm, the firing behavior of individual neurons (excitatory and inhibitory) is close to a Poisson process. Short-lived gamma oscillations are produced at the network level especially in the transient synchrony regime^41^.

In this formalism, it is possible to approximate the Poisson statistics by Gaussian statistics for firings in any time interval. This leads to the following activity of the excitatory population defined as *E*(*t*) = *k*(*t*)/*N*_*E*_^41^:

11 $$\frac{dE(t)}{dt}=-\,{\alpha }_{E}E(t)+(1-E(t)){\beta }_{E}f({s}_{{\rm{E}}}(t))+{I}_{E}(t)$$

Similarly for inhibitory neurons, we have:

12 $$\frac{dI(t)}{dt}=-\,{\alpha }_{I}I(t)+(1-I(t)){\beta }_{I}f({s}_{I}(t))+{I}_{I}(t)$$

with noise sources with time-dependent variances given by

$${I}_{E}(t)=\sqrt{\frac{(1-E(t)){\beta }_{E}f({s}_{E}(t))+{\alpha }_{E}E(t)}{{N}_{E}}}{\eta }_{E}(t)\,{\rm{and}}\,{I}_{I}(t)=\sqrt{\frac{(1-I(t)){\beta }_{I}f({s}_{I}(t))+{\alpha }_{I}I(t)}{{N}_{I}}}{\eta }_{I}(t).$$

Here *η*_*E*,*I*_(*t*) are Gaussian white noises satisfying:

$$\langle {\eta }_{i}(t)\rangle =0,\,\langle {\eta }_{i}(t){\eta }_{j}(t^{\prime} )\rangle ={\delta }_{ij}\delta (t-t^{\prime} )\,i,\,j=\{E,\,I\}.$$

According to the Linear Noise Approximation (LNA), if *N*_*E*_ and *N*_*I*_ are quite large but stochastic effects are still important, one may apply a further Gaussian approximation. The activities (*k*, *l*) can then be represented as the sum of a deterministic component (*E*_0_, *I*_0_) scaled by the population sizes and stochastic perturbations $({\tilde{V}}_{E}(t),{\tilde{V}}_{I}(t))$ scaled by square root of the population sizes^41^. We then have

13 $$E(t)={E}_{0}(t)+\frac{1}{\sqrt{{N}_{E}}}{\tilde{V}}_{E},\,I(t)={I}_{0}(t)+\frac{1}{\sqrt{{N}_{I}}}{\tilde{V}}_{I}(t)$$

where *E*_0_(*t*) and *I*_0_(*t*) are solutions of the deterministic version of Eqs. 11 and 12 above:

14 $$\frac{d{E}_{0}(t)}{dt}=-\,{\alpha }_{E}{E}_{0}(t)+(1-{E}_{0}(t)){\beta }_{E}f({s}_{{E}_{0}}(t))$$

15 $$\frac{d{I}_{0}(t)}{dt}=-\,{\alpha }_{I}{I}_{0}(t)+(1-{I}_{0}(t)){\beta }_{I}f({s}_{{I}_{0}}(t))$$

with

$${s}_{{E}_{0}}={W}_{ee}{E}_{0}-{W}_{ei}{I}_{0}+{h}_{E},\,{s}_{{I}_{0}}={W}_{ie}{E}_{0}-{W}_{ii}{I}_{0}+{h}_{I}\,.$$

We focus on oscillations induced by noise, for which Eqs. 14 and 15 must admit a stable equilibrium or “fixed” point (i.e. its complex eigenvalues have negative real part). This fixed point is the solution of

16 $$\begin{array}{rcl}0 & = & -{a}_{E}{E}_{0}^{\ast }+(1-{E}_{0}^{\ast }){\beta }_{E}f({s}_{{E}_{0}^{\ast }})\\ 0 & = & -{\alpha }_{I}{I}_{0}^{\ast }+(1-{I}_{0}^{\ast }){\beta }_{I}f({s}_{{I}_{0}^{\ast }}).\end{array}$$

After a transient, the deterministic solution (*E*_0_(*t*), *I*_0_(*t*)) converges to the fixed point (*E*_0_^*^, *I*_0_^*^) and the LNA becomes:

17 $$E(t)={E}_{0}^{\ast }+\frac{1}{\sqrt{{N}_{E}}}{\tilde{V}}_{E}(t),\,I(t)={I}_{0}^{\ast }+\frac{1}{\sqrt{{N}_{I}}}{\tilde{V}}_{I}(t)\,.$$

Replacing Eq. 17 into Eqs. 11 and 12 and keeping the terms of order ${\mathscr{O}}(\sqrt{{N}_{E}})$ and ${\mathscr{O}}(\sqrt{{N}_{I}})$, the dynamics of fluctuations around the equilibrium point are obtained:

$$\frac{d{\tilde{V}}_{E}(t)}{dt}={A}_{11}{\tilde{V}}_{E}(t)+{A}_{12}{\tilde{V}}_{I}(t)+{\sigma }_{E}{\eta }_{E}(t)$$

$$\frac{d{\tilde{V}}_{I}(t)}{dt}={A}_{21}{\tilde{V}}_{E}(t)+{A}_{22}{\tilde{V}}_{I}(t)+{\sigma }_{I}{\eta }_{I}(t)\,.$$

In terms of all the biophysical parameters of the original nonlinear stochastic spiking E-I network, the seven parameters governing these fluctuations around the equilibrium are given by:

$${A}_{11}=-\,{\alpha }_{E}-{\beta }_{E}f({s}_{{E}_{0}^{\ast }})+(1-{E}_{0}^{\ast }){W}_{ee}{\beta }_{E}{f}^{{\rm{^{\prime} }}}({s}_{{E}_{0}^{\ast }})=-\,\frac{{\alpha }_{E}}{1-{E}_{0}^{\ast }}+{\alpha }_{E}{E}_{0}^{\ast }{\textstyle [}1-\frac{{\alpha }_{E}{E}_{0}^{\ast }}{(1-{E}_{0}^{\ast }){\beta }_{E}}{\textstyle ]}{W}_{ee}$$

$${A}_{12}=-\,(1-{E}_{0}^{\ast }){W}_{ei}{\beta }_{E}{f}^{{\rm{^{\prime} }}}({s}_{{E}_{0}^{\ast }}){c}_{EI}=-\,{c}_{EI}{\alpha }_{E}{E}_{0}^{\ast }{\textstyle [}1-\frac{{\alpha }_{E}{E}_{0}^{\ast }}{(1-{E}_{0}^{\ast }){\beta }_{E}}{\textstyle ]}{W}_{ei}$$

$${A}_{21}=(1-{I}_{0}^{\ast }){W}_{ie}{\beta }_{I}{f}^{{\rm{^{\prime} }}}({s}_{{I}_{0}^{\ast }}){c}_{EI}^{-1}={c}_{EI}^{-1}{\alpha }_{I}{I}_{0}^{\ast }{\textstyle [}1-\frac{{\alpha }_{I}{I}_{0}^{\ast }}{(1-{I}_{0}^{\ast }){\beta }_{I}}{\textstyle ]}{W}_{ie}$$

$${A}_{22}=-\,{\alpha }_{I}-{\beta }_{I}f({s}_{{I}_{0}^{\ast }})-(1-{I}_{0}^{\ast }){W}_{ii}{\beta }_{I}{f}^{{\rm{^{\prime} }}}({s}_{{I}_{0}^{\ast }})=-\,\frac{{\alpha }_{I}}{1-{I}_{0}^{\ast }}-{\alpha }_{I}{I}_{0}^{\ast }{\textstyle [}1-\frac{{\alpha }_{I}{I}_{0}^{\ast }}{(1-{I}_{0}^{\ast }){\beta }_{I}}{\textstyle ]}{W}_{ii}$$

$${\sigma }_{E}=\sqrt{{\alpha }_{E}{E}_{0}^{\ast }+(1-{E}_{0}^{\ast }){\beta }_{E}f({s}_{{E}_{0}^{\ast }})}$$

$${\sigma }_{I}=\sqrt{{\alpha }_{I}{I}_{0}^{\ast }+(1-{I}_{0}^{\ast }){\beta }_{I}f({s}_{{I}_{0}^{\ast }})}$$

$${c}_{EI}=\sqrt{\frac{{N}_{E}}{{N}_{I}}}.$$

Using the fixed point equations, the noise intensities can be rewritten as ${\sigma }_{E}=\sqrt{2{\alpha }_{E}{E}_{0}^{\ast }}$ and ${\sigma }_{I}=\sqrt{2{\alpha }_{I}{I}_{0}^{\ast }}$. Therefore we obtain linear equations driven by noise which represent the LFP dynamics ${\tilde{V}}_{E}$ and ${\tilde{V}}_{I}$. Changing one parameter, such as the strength of connectivity of I cells onto E cells *W*_*ei*_, will change a number of these parameters as well as the fixed points. In turn we will see below that these changes impact only two “master parameters” that govern the envelope dynamics.

### Linear analysis

We consider the linear stochastic Eqs. 1 and 2 and first consider the deterministic case *σ*_*E*_ = *σ*_*I*_ = 0. The associated noise-free linear system is written in the following matrix form:

$$\frac{d{V}^{0}(t)}{dt}=A{V}^{0}(t)$$

where

$${V}^{0}(t)={\textstyle [}\begin{array}{c}{V}_{E}^{0}(t)\\ {V}_{I}^{0}(t)\end{array}{\textstyle ]}\,and\,A={\textstyle [}\begin{array}{cc}{A}_{11} & {A}_{12}\\ {A}_{21} & {A}_{22}\end{array}{\textstyle ]}.$$

We look for a trial solution in the form:

$${\textstyle [}\begin{array}{c}{V}_{E}^{0}(t)\\ {V}_{I}^{0}(t)\end{array}{\textstyle ]}={\textstyle [}\begin{array}{c}{\mathop{B}\limits^{ \sim }}_{E}\\ {\mathop{B}\limits^{ \sim }}_{I}\end{array}{\textstyle ]}{e}^{\lambda t}.$$

where ${\tilde{B}}_{E}={B}_{E}{e}^{j{\theta }_{E}}$ and ${\tilde{B}}_{I}={B}_{I}{e}^{j{\theta }_{I}}$. The eigenvalue *λ* of the associated matrix *A* is found by substituting the trial solution into the linear system, yielding

$$\frac{{\tilde{B}}_{E}}{{\tilde{B}}_{I}}=\frac{-{A}_{12}}{{A}_{11}-\lambda }=-\frac{{A}_{22}-\lambda }{{A}_{21}}$$

The second equality leads to

$$\lambda =\frac{1}{2}({A}_{11}+{A}_{22})\pm \frac{j}{2}\sqrt{-{({A}_{11}-{A}_{22})}^{2}-4{A}_{12}{A}_{21}}.$$

We rewrite the eigenvalue in the compact form

18 $$\lambda =-\,\nu \pm j{\omega }_{0}$$

with

$$\nu =-\,\frac{{A}_{11}+{A}_{22}}{2},\,{\omega }_{0}=\frac{1}{2}\sqrt{-{({A}_{11}-{A}_{22})}^{2}-4{A}_{12}{A}_{21}}\,\,and\,\,j=\sqrt{-1}.$$

This leads to the exact expression of the complex amplitude ratio and phase difference between the excitatory and inhibitory LFPs:

$$\frac{{B}_{E}}{{B}_{I}}=\sqrt{\frac{{A}_{12}}{-{A}_{21}}}=\sqrt{\frac{{c}_{EI}{\alpha }_{E}{E}_{0}^{\ast }[1-\frac{{\alpha }_{E}{E}_{0}^{\ast }}{(1-{E}_{0}^{\ast }){\beta }_{E}}]{W}_{EI}}{{c}_{EI}^{-1}{\alpha }_{I}{I}_{0}^{\ast }[1-\frac{{\alpha }_{I}{I}_{0}^{\ast }}{(1-{I}_{0}^{\ast }){\beta }_{I}}]{W}_{IE}}}\approx \sqrt{\frac{{N}_{I}{W}_{IE}}{{N}_{E}{W}_{EI}}}.$$

and

$$\delta ={\theta }_{E}-{\theta }_{I}={\textstyle \{}\begin{array}{cc}\arctan {\textstyle (}\frac{2{\omega }_{0}}{{A}_{11}-{A}_{22}}{\textstyle )}\,if & {A}_{11}-{A}_{22} > 0\\ \pi +\arctan {\textstyle (}\frac{2{\omega }_{0}}{{A}_{11}-{A}_{22}}{\textstyle )} & otherwise.\end{array}$$

Note that in the absence of noise, the time-dependent amplitudes both go to zero exponentially with characteristic time *ν*^−1^. One can nevertheless compute the ratio of amplitudes as above. However, in the presence of noise, one can compute the ratio from simulated time series using the analytic signal technique. The amplitudes ratio and the phase difference are obtained by the following approximations:

19 $$\alpha =\frac{{B}_{I}}{{{\rm{B}}}_{E}}\approx {\textstyle \langle }\frac{Env[{\mathop{V}\limits^{ \sim }}_{I}(t)]}{Env[{\mathop{V}\limits^{ \sim }}_{E}(t)]}{\textstyle \rangle }\,\,and\,\,\delta ={\theta }_{E}-{\theta }_{I}\approx {\textstyle \langle }Arg[{\mathop{V}\limits^{ \sim }}_{E}(t)]-Arg[{\mathop{V}\limits^{ \sim }}_{I}(t)]{\textstyle \rangle }\,.$$

Here 〈.〉 can be considered a time average of the stochastic process in Eq. 1. *Env* is defined as the envelope of the analytic signal associated with the LFP. For example, the analytic signal corresponding to *V*_*E*_(*t*) is *V*_*E*_(*t*) + *jH*[*V*_*E*_(*t*)], with the Hilbert transform *H* defined as

20 $$H[x]=\frac{1}{\pi }P{\int }_{-\infty }^{\infty }\,\frac{x(\tau )}{t-\tau }d\tau $$

where *P* signifies the Cauchy principal value. The envelope of the stochastic signal is then $Env[{V}_{E}]=\sqrt{{V}_{E}^{2}+{H}^{2}[{V}_{E}]}$. Likewise, the phase angle of the analytic signal is defined as $Arg[{V}_{E}]=\arctan [H[{V}_{E}]/{V}_{E}]$.

The transition between the transient and high synchrony regimes happens when the real part of the eigenvalue is zero. This condition is expressed as

21 $$\nu =\,-\,\frac{{\alpha }_{E}}{1-{E}_{0}^{\ast }}+{\alpha }_{E}{E}_{0}^{\ast }{\textstyle [}1-\frac{{\alpha }_{E}{E}_{0}^{\ast }}{(1-{E}_{0}^{\ast }){\beta }_{E}}{\textstyle ]}{W}_{ee}-\frac{{\alpha }_{I}}{1-{I}_{0}^{\ast }}-{\alpha }_{I}{I}_{0}^{\ast }{\textstyle [}1-\frac{{\alpha }_{I}{I}_{0}^{\ast }}{(1-{I}_{0}^{\ast }){\beta }_{I}}{\textstyle ]}{W}_{ii}=0.$$

We use this expression to plot Fig. 4(Left panel). For Fig. 4(Right panel), we first use Eq. 9 to shift from the self-connectivity parameters to the (*W*_*ei*_, *W*_*ie*_) plane. We next derive an expression for the dynamics governing the time evolution of the envelopes of the excitatory and inhibitory stochastic processes themselves.

### Stochastic Averaging Method (SAM)

Taking into account the constant ratio of envelopes and constant phase difference (see our three assumptions early in the Results section), the expression of the excitatory and inhibitory LFPs are given by

22 $${V}_{E}(t)={Z}_{E}(t)\cos {\textstyle (}{\omega }_{0}t+{\varphi }_{E}(t){\textstyle )}\,\,\,\,and\,\,\,\,{V}_{I}(t)=\alpha {Z}_{E}(t)\cos {\textstyle (}{\omega }_{0}t+{\varphi }_{E}(t)-\delta {\textstyle )}\,.$$

We plug these expressions into the linear stochastic equations Eq. 1 and rewrite the resulting equations in terms of variables *Z*_*E*_ and *ϕ*_*E*_ as follows:

23 $${\dot{Z}}_{E}(t)={f}_{1}({Z}_{E},{\varphi }_{E})+{g}_{1}({Z}_{E},{\varphi }_{E},{\eta }_{E},{\eta }_{I})$$

24 $${\dot{\varphi }}_{E}(t)={f}_{2}({Z}_{E},{\varphi }_{E})+{g}_{2}({Z}_{E},{\varphi }_{E},{\eta }_{E},{\eta }_{I})$$

with

25 $$\begin{array}{ccc}{f}_{1}({Z}_{E},{\varphi }_{E}) & = & \frac{{Z}_{E}}{\alpha sin\delta }{\textstyle [}\alpha (\,-\,{\omega }_{0}sin({\omega }_{0}t+{\varphi }_{E})+{A}_{11}\,\cos ({\omega }_{0}t+{\varphi }_{E})\\  &  & +\alpha {A}_{12}\,\cos ({\omega }_{0}t+{\varphi }_{E}-\delta ))\sin ({\omega }_{0}t+{\varphi }_{E}-\delta ))\\  &  & -(\alpha {\omega }_{0}sin({\omega }_{0}t+{\varphi }_{E}-\delta )+{A}_{21}\,\cos ({\omega }_{0}t+{\varphi }_{E})\\  &  & +\alpha {A}_{22}\,\cos ({\omega }_{0}t+{\varphi }_{E}-\delta ))\sin ({\omega }_{0}t+{\varphi }_{E}){\textstyle ]}\end{array}$$

26 $$\begin{array}{ccc}{f}_{2}({Z}_{E},{\varphi }_{E}) & = & \frac{1}{\alpha sin\delta }{\textstyle [}\alpha (-\,{\omega }_{0}sin({\omega }_{0}t+{\varphi }_{E})+{A}_{11}\,\cos ({\omega }_{0}t+{\varphi }_{E})\\  &  & +\alpha {A}_{12}\,\cos ({\omega }_{0}t+{\varphi }_{E}-\delta ))\cos ({\omega }_{0}t+{\varphi }_{E}-\delta )\\  &  & -(\alpha {\omega }_{0}sin({\omega }_{0}t+{\varphi }_{E}-\delta )+{A}_{21}\,\cos ({\omega }_{0}t+{\varphi }_{E})\\  &  & +\alpha {A}_{22}\,\cos ({\omega }_{0}t+{\varphi }_{E}-\delta ))\cos ({\omega }_{0}t+{\varphi }_{E}){\textstyle ]}\end{array}$$

and

27 $${g}_{1}({Z}_{E},{\varphi }_{E},{\eta }_{E},{\eta }_{I})=\frac{1}{\alpha \,\sin \,\delta }{\textstyle [}\,-\,{\sigma }_{E}\alpha \,\sin ({\omega }_{0}t+{\varphi }_{E}-\delta ){\eta }_{E}+{\sigma }_{I}\,\sin ({\omega }_{0}t+{\varphi }_{E}){\eta }_{I}{\textstyle ]}$$

28 $${g}_{2}({Z}_{E},{\varphi }_{E},{\eta }_{E},{\eta }_{I})=\frac{1}{\alpha {Z}_{E}\,\sin \,\delta }{\textstyle [}{\sigma }_{I}\,\cos ({\omega }_{0}t+{\varphi }_{E}){\eta }_{I}-{\sigma }_{E}\alpha \,\cos ({\omega }_{0}t+{\varphi }_{E}-\delta ){\eta }_{E}{\textstyle ]}\,.$$

The equations above can be written in a more compact form as

29 $$\dot{X}(t)=f(X)+g(X,\eta )\,,$$

with the following 2 × 1 matrix definitions: $$X={\textstyle [}\begin{array}{c}{Z}_{E}\\ {\varphi }_{E}\end{array}{\textstyle ]}$$, $$f={\textstyle [}\begin{array}{c}{f}_{1}\\ {f}_{2}\end{array}{\textstyle ]}$$, $$g={\textstyle [}\begin{array}{c}{g}_{1}\\ {g}_{2}\end{array}{\textstyle ]}$$ and $$\eta ={\textstyle [}\begin{array}{c}{\eta }_{E}\\ {\eta }_{I}\end{array}{\textstyle ]}\,.$$ The stochastic averaging method says that, under certain conditions (usually met for regular functions like f and g), the above system of two stochastic differential equations can be approximated by the following 2-dimensional Markov process^93^:

30 $$dX(t)=m(X)dt+h(X)dW(t),$$

where *m* is a 2 × 1 matrix, *h* is a 2 × 2 matrix and *W*(*t*) denotes a 2-dimensional vector of independent Wiener processes with unit variance. Also, *m* and *h* are respectively *O*(*ε*^2^) and *O*(*ε*) functions (ε is an infinitesimal number) defined as:

31 $$m={T}^{av}{\textstyle (}E\{f\}+{\int }_{-{\rm{\infty }}}^{0}\,E{\textstyle \{}{{\textstyle (}\frac{{\rm{\partial }}g}{{\rm{\partial }}X}{\textstyle )}}_{t}{(g)}_{t+\tau }{\textstyle \}}d\tau {\textstyle )}$$

32 $$h{h}^{{\rm{^{\prime} }}}={T}^{av}{\textstyle (}{\int }_{-{\rm{\infty }}}^{{\rm{\infty }}}\,E{\textstyle \{}{(g)}_{t}{({g}^{{\rm{^{\prime} }}})}_{t+\tau }{\textstyle \}}d\tau {\textstyle )}$$

here (') denotes transposition, and ${{\textstyle (}\frac{{\rm{\partial }}g}{{\rm{\partial }}X}{\textstyle )}}_{t}$ is a 2 × 2 Jacobian matrix. Moreover, *E*. denotes the expectation operator and *T*^*av*^ is the time averaging operator defined by

33 $${T}^{av}(.)=\frac{1}{{T}_{0}}{\int }_{t0}^{t0+T0}\,(.)dt$$

where ${T}_{0}=\frac{2\pi }{{\omega }_{0}}$ is the period of a gamma oscillation cycle. When evaluating the expectations in the stochastic averages formula, the elements of *X* are treated as constants in time. A somewhat lengthy calculation leads to the resulting Markov processes for the LFP envelope and phase:

34 $$d{Z}_{E}(t)={\textstyle (}-\nu {Z}_{E}(t)+\frac{D}{2{Z}_{E}(t)}{\textstyle )}dt+\sqrt{D}d{W}_{1}(t)$$

35 $$d{\varphi }_{E}(t)=\frac{\sqrt{D}}{{Z}_{E}(t)}d{W}_{2}(t)$$

where

36 $$D=-\,\frac{{A}_{12}}{2{\omega }_{0}^{2}}{\textstyle (}\,-\,{A}_{12}{\sigma }_{I}^{2}+{A}_{21}{\sigma }_{E}^{2}{\textstyle )}\,.$$

Note that the coefficient *D* is zero when both the excitatory and/or inhibitory noise intensities *σ*_*E*_ and *σ*_*I*_ are zero. One can call it a noise-induced coefficient in the drift part of the stochastic differential equation for the envelope.

For computational purposes, the envelope and phase equations above can be rewritten using two independent Ornstein-Uhlenbeck (OU) processes as:

37 $$d{E}_{1}(t)=-\,\nu {E}_{1}(t)dt+\sqrt{D}d{W}_{1}(t)$$

38 $$d{E}_{2}(t)=-\,\nu {E}_{2}(t)dt+\sqrt{D}d{W}_{2}(t)$$

from which we can extract the envelope and phase:

39 $${Z}_{E}(t)=\sqrt{{E}_{1}^{2}(t)+{E}_{2}^{2}(t)}\,\,{\varphi }_{E}(t)=\arctan {\textstyle (}\frac{{E}_{2}(t)}{{E}_{1}(t)}{\textstyle )}.$$

These quantities satisfy the differential equations for *Z*_*E*_ and *ϕ*_*E*_ above. The envelope and phase processes are then the envelope and phase of two independent Ornstein-Uhlenbeck processes with the same parameters. Our simulations actually use these two OU processes, rather than the *Z*_*E*_ − Φ_*E*_ equations above, in order to avoid the occurrence of negative values of *Z*_*E*_. The corresponding equations for the inhibitory population are obtained from these ones by using the ratio and phase difference factors in Eq. 19. This ratio and phase difference are to be interpreted as constant averaged quantities; they will fluctuate around these quantities in any finite realization.

**Figure 8:**
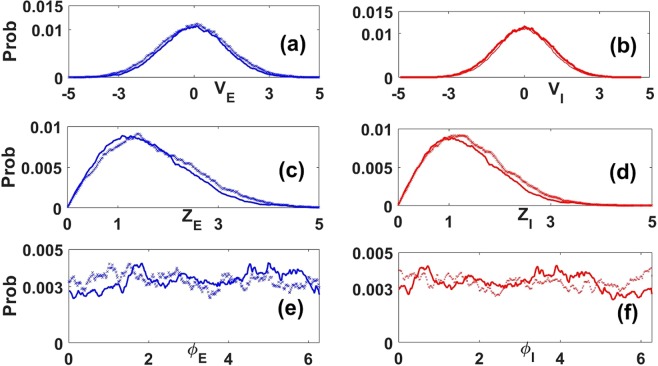
Probability distributions of LFPs ((**a**) and (**b**)), envelopes ((**c**) and (**d**)) and phases ((**e**) and (**f**)) computed from LNA versus SAM. Solid lines are distributions computed from LNA Eqs. 1 and 2, while crossed lines are those computed from SAM Eqs. 5 and 6 and Eq. 22. Blues lines (**a**), (**c**) and (**e**) corresponds to excitatory components and reds lines (**b**), (**d**) and (**f**) to Inhibitory ones. We can observe good matching between LNA and SAM dynamics, which shows that the dynamics obtained from SAM are statistically similar to those in the LNA. The parameters are taken in Table 1.

Probability distributions in Fig. 8 show that the dynamics obtained from SAM are statistically equivalent to those of the LNA. This suggests that our SAM is an appropriate framework for envelope and phase dynamics of bursty gamma oscillations.

### Probability density and Mean First Passage Times (MFPT)

For simplicity, we consider the envelope of the excitatory population and denote it *z*(*t*). The envelope process with its initial condition is given by (see Eq. 35):

40 $$\begin{array}{ccc}dz(t) & = & {\textstyle (}-\nu z(t)+\frac{D}{2z(t)}{\textstyle )}dt+\sqrt{D}dW(t)\\ z(0) & = & {z}_{0}.\end{array}$$

The associated Fokker-Planck equation for the probability density of *z*(*t*), conditioned on the initial condition, is given by

41 $$\frac{{\rm{\partial }}P(z,t|{z}_{0},0)}{{\rm{\partial }}t}=-\,\frac{{\rm{\partial }}}{{\rm{\partial }}z}{\textstyle [}-\nu z+\frac{D}{2z}{\textstyle ]}P(z,t|{z}_{0},0)+\frac{D}{2}\frac{{{\rm{\partial }}}^{2}P(z,t|{z}_{0},0)}{{\rm{\partial }}{z}^{2}}\,.$$

In the stationary limit, this reduces to the differential equation

$$-\frac{d}{dz}{\textstyle [}-\nu z+\frac{D}{2z}{\textstyle ]}P(z)+\frac{D}{2}\frac{{d}^{2}P(z)}{d{z}^{2}}=0\,.$$

The stationary probability function then reads

42 $$P(z)={\textstyle (}\frac{2\nu }{D}{\textstyle )}z\exp {\textstyle (}-\frac{\nu }{D}{z}^{2}{\textstyle )}.$$

The peak value is obtained by imposing

43 $${\frac{dP(z)}{dz}{\textstyle |}}_{z={z}^{\ast }}=0$$

which leads to

44 $${z}^{\ast }=\sqrt{\frac{D}{2\nu }}\,.$$

Note that z^*^ ≡ *R* in the main text. Properties such as the mean and standard deviation of *z*(*t*) can be easily computed from the stationary probability density function, and are known for decades as properties of the Rayleigh distribution^58^. The mean and the standard deviation are given by:

$$E[z]=\sqrt{\frac{\pi }{2}}{z}^{\ast }\,and\,std[z]=\sqrt{E[{z}^{2}]-E{[z]}^{2}}={z}^{\ast }{\textstyle (}\sqrt{\frac{4-\pi }{2}}{\textstyle )}.$$

**Figure 9:**
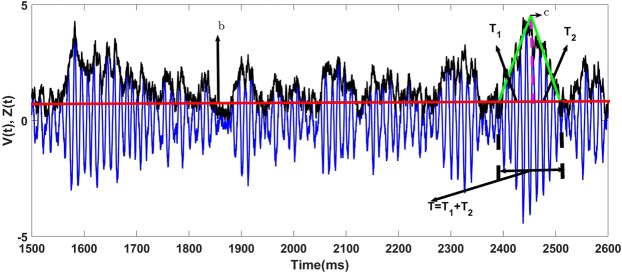
Typical burst duration following our approach. Computation of the mean burst duration. The green bars show the increase and decrease of the envelope process in black. The vertical dashed magenta line shows the separation between the two mean first passage times. The red bar sets the value of the threshold *b*. A typical burst is the epoch during which the envelope stays above the threshold and the burst duration is the corresponding time.

A burst is defined as an epoch during which the envelope process stays above a particular threshold (see Fig. 9). A full theoretical treatment leading to the density of such epochs - known as residence times - is mathematically very involved and beyond the scope of this paper. Rather, here we resort to an approximate derivation of the properties of these epochs that yields some analytical insight into their parameter dependence. The burst duration can be seen to correspond roughly to the time the amplitude process spends reaching its maximum value after crossing the threshold from below, plus the time it spends from this maximum value until it crosses the threshold again but from above (see Fig. 9). These two durations can be expressed distinctly by their associated Mean First Passage Times (MFPT). Generally, the MFPT from an initial condition *z*_0_ to a specific border of an interval *A* where the amplitude process is confined is given by^57^:

45 $$T({z}_{0})=-\,{\int }_{0}^{\infty }\,t\frac{\partial }{\partial t}\mathop{\int }\limits_{A}\,dzP(z,t|{z}_{0},0)dt.$$

The MFPT also satisfies the following first-order differential equation^57^:

46 $${\textstyle (}-\,\nu {z}_{0}+\frac{D}{2{z}_{0}}{\textstyle )}\frac{dT({z}_{0})}{d{z}_{0}}+\frac{D}{2}\frac{{d}^{2}T({z}_{0})}{{d}^{2}{z}_{0}}=-\,1.$$

In our case, we define the interval where the process lies to be *A* = [*b*, *c*] where *b* is the threshold defining the start and the end of a burst, while *c* is a “typical” maximum value that the envelope process can attain during that burst. During the period when the envelope increases towards its maximum, an absorbing boundary condition is imposed at *c* leading to *T*_1_(*c*) = 0, and a reflecting boundary condition is imposed at *b*, given by ${\frac{d{T}_{1}({z}_{0})}{d{z}_{0}}{\textstyle |}}_{{z}_{0}=b}=0$. This results in the following expression for the “first” MFPT on the way up:

47 $${T}_{1}({z}_{0})=\frac{{e}^{(-\frac{\nu }{D}{b}^{2})}}{2\nu }{\textstyle [}Ei{\textstyle (}\frac{\nu }{D}{c}^{2}{\textstyle )}-Ei{\textstyle (}\frac{\nu }{D}{z}_{0}^{2}{\textstyle )}{\textstyle ]}-\frac{1}{\nu }\,\log {\textstyle (}\frac{c}{{z}_{0}}{\textstyle )}$$

where *Ei* is the integral exponential function defined as^94^:

$$Ei(x)=-\,\int \frac{{e}^{x}}{x}dx.$$

Further, assigning the threshold value *b* to the initial condition of an above-threshold epoch, then the first MFPT is given by

48 $${T}_{1}(b)=\frac{{e}^{(-\frac{\nu }{D}{b}^{2})}}{2\nu }{\textstyle [}Ei{\textstyle (}\frac{\nu }{D}{c}^{2}{\textstyle )}-Ei{\textstyle (}\frac{\nu }{D}{b}^{2}{\textstyle )}{\textstyle ]}-\frac{1}{\nu }\,\log {\textstyle (}\frac{c}{b}{\textstyle )}.$$

To compute the time interval for the process to leave its maximum value and cross the threshold from above, a reflecting boundary condition is now set at *c*, which translates into $\frac{d{T}_{2}({z}_{0})}{d{z}_{0}}{{\textstyle |}}_{{z}_{0}=c}=0$, and an absorbing condition at *b*, *T*_2_(*b*) = 0. The associated “second” MFPT is then given by

49 $${T}_{2}({z}_{0})=\frac{{e}^{(-\frac{\nu }{D}{c}^{2})}}{2\nu }{\textstyle [}Ei{\textstyle (}\frac{\nu }{D}{b}^{2}{\textstyle )}-Ei{\textstyle (}\frac{\nu }{D}{z}_{0}^{2}{\textstyle )}{\textstyle ]}+\frac{1}{\nu }\,\log {\textstyle (}\frac{{z}_{0}}{b}{\textstyle )}.$$

We now assign *z*_0_ = *c* and the second mean duration is

50 $${T}_{2}(c)=\frac{{e}^{(-\frac{\nu }{D}{c}^{2})}}{2\nu }{\textstyle [}Ei{\textstyle (}\frac{\nu }{D}{b}^{2}{\textstyle )}-Ei{\textstyle (}\frac{\nu }{D}{c}^{2}{\textstyle )}{\textstyle ]}+\frac{1}{\nu }\,\log {\textstyle (}\frac{c}{b}{\textstyle )}.$$

Therefore, the approximated burst duration is given by *T* = *T*_1_(*b*) + *T*_2_(*c*), which simplifies to

51 $$T={\textstyle (}\frac{1}{2\nu }{\textstyle )}{\textstyle [}\exp {\textstyle (}-\frac{1}{2}{{\textstyle (}\frac{b}{R}{\textstyle )}}^{2}{\textstyle )}-\exp {\textstyle (}-\frac{1}{2}{{\textstyle (}\frac{c}{R}{\textstyle )}}^{2}{\textstyle )}{\textstyle ]}{\textstyle [}Ei{\textstyle (}-\frac{1}{2}{{\textstyle (}\frac{c}{R}{\textstyle )}}^{2}{\textstyle )}-Ei{\textstyle (}-\frac{1}{2}{{\textstyle (}\frac{b}{R}{\textstyle )}}^{2}{\textstyle )}{\textstyle ]},$$

(Where we have used the relation $R=\sqrt{\frac{D}{2\nu }}$).

While the choice of *b* and *c* are arbitrary, we found that satisfactory estimates of mean burst durations followed from choices that made intuitive sense. Specifically, we chose the threshold to be equal to half the median of the envelope density *P*(*z*); this corresponds to setting $b=R\sqrt{\mathrm{ln}(2)/2}\approx 0.59R$. We choose the value of *c* as the mean of *P*(*z*), i.e. $R\sqrt{\pi /2}$ plus one standard deviation $R\sqrt{(4-\pi )/2}$:

$$c=R{\textstyle (}\sqrt{\frac{\pi }{2}}+\sqrt{\frac{4-\pi }{2}}{\textstyle )}.$$

This approximate analysis provides an estimate of the mean burst duration as a function of the synchronization parameter *R*. One could also choose a threshold that does not depend on *R* or any other parameter, but that would yield no bursts for smaller *R* values, even though close inspection of the smaller envelope reveals burstiness at the smaller scale.
